# DNA Methylation in the Exon 1 Region and Complex Regulation of Twist1 Expression in Gastric Cancer Cells

**DOI:** 10.1371/journal.pone.0145630

**Published:** 2015-12-22

**Authors:** Ayuna Sakamoto, Yoshimitsu Akiyama, Shu Shimada, Wei-Guo Zhu, Yasuhito Yuasa, Shinji Tanaka

**Affiliations:** 1 Department of Molecular Oncology, Graduate School of Medical and Dental Sciences, Tokyo Medical and Dental University, Tokyo, Japan; 2 Department of Biochemistry and Molecular Biology, Peking University Health Science Center, Beijing, 100191, China; Pohang University of Science and Technology (POSTECH), REPUBLIC OF KOREA

## Abstract

Twist1 overexpression is frequently observed in various cancers including gastric cancer (GC). Although DNA methylation of the *Twist1* gene has been reported in cancer cells, the mechanisms underlying transcriptional activation remain uncertain. In this study, we first examined epigenetic alterations of the *Twist1* using *Twist1* transcription-positive and -negative cell lines that are derived from our established diffuse-type GC mouse model. Treatment with a DNA demethylation agent 5-aza-dC re-activated Twist1 expression in Twist1 expression-negative GC cells. According to methylation-specific PCR and bisulfite sequencing analysis, methylation at the CpG-rich region within *Twist1* coding exon 1, rather than its promoter region, was tightly linked to transcriptional silencing of the *Twist1* expression in mouse GC cells. Chromatin immunoprecipitation assays revealed that active histone mark H3K4me3 was enriched in Twist1 expression-positive cells, and inactive histone mark H3K9me3 was enriched in Twist1 expression-negative cells. The expression levels of *Suv39h1* and *Suv39h2*, histone methyltransferases for H3K9me3, were inversely correlated with *Twist1* expression, and knockdown of *Suv39h1* or *Suv39h2* induced *Twist1* expression. Moreover, Sp1 transcription factor bound to the exon 1 CpG-rich region in Twist1 expression-positive cell lines, and *Twist1* expression was diminished by mithramycin, which that interferes with Sp1 binding to CpG-rich regulatory sequences. Our studies suggested that the *Twist1* transcription in GC cells might be regulated through potential cooperation of DNA methylation, histone modification in complex with Sp1 binding to CpG-rich regions within the exon 1 region.

## Introduction

Twist1, acting as a basic helix-loop-helix transcription factor, directly binds to E-box elements (NCANNTGN) on specific target genes [1]. Twist1 is widely known to be essential for mesoderm formation during early embryonic development of drosophila and mouse [2, 3]. In human cancers, ectopic Twist1 expression is reported to be associated with malignant progression, invasion, epithelial-to-mechencymal transition, metastasis and stemness, indicating potential oncogenic functions of Twist1 [4–6]. Thus, it is very important to clarify the transcriptional regulatory mechanisms for Twist1 in cancer cells.

Epigenetic changes, such as DNA methylation and histone modification, are tightly linked to gene silencing [7–9]. Although epigenetic alterations at the CGI (CpG island) promoter region of tumor suppressor genes (TSGs) are well-known to be associated with their gene inactivation [10], the mechanisms of epigenetic regulation of oncogenes are poorly understood. Several groups have shown that *Twist1* was re-activated by treatment with a de-methylation drug, 5-aza-2’-deoxycitidine (5-aza-dC), in cancer cells [11, 12]. Aberrant DNA methylation at the CGI promoter in human *Twist1* has been frequently detected in primary cancers including of gastric cancer (GC) [11–14]. Nevertheless, there are a lot of findings that DNA methylation at the *Twist1* promoter region is not correlated with expression of the gene in various cancers [12, 14]. While *Twist1* methylation may be a useful biomarker to predict the clinical outcomes as to recurrence and survival in patients with cancers [12, 13, 15], it remains controversial whether or not the *Twist1* methylation at the promoter region leads to silencing of the gene.

Transcriptional regulation of genes is correlated with lysine (K) methylation patterns in the histone tail that consist of three different lysine methylation states (mono-, di- and tri-). Tri-methylation of H3K4 (H3K4me3) is related to gene activation, and that of H3K9me3 and H3K27me3 to gene repression [7–9]. Although these three histone H3-methyaltion patterns are linked to CGI methylation as well, the transcriptional regulatory mechanisms for *Twist1* underlying histone modification are poorly understood in cancer cells.

GC is the second most frequent cause of death from cancer in the world [16]. GC is classified into two main histological types; diffuse and intestinal, which are two distinct carcinogenic pathways [17]. Diffuse-type gastric cancer (DGC) is known to show frequent invasion and metastasis, resulting in a poor prognosis [18]. Loss of E-cadherin and p53 has been reported in diffuse-type gastric carcinogenesis [19–21]. Several reports demonstrated frequent overexpression of Twist1 in human DGCs [22, 23].

We previously engineered a double conditional knockout (DCKO) mouse line as to E-cadherin and p53, which are specifically inactivated in the stomach [19]. All the DCKO mice developed fatal DGCs within a year, the phenotypes being high invasiveness and frequent metastasis to lymph nodes. It is noteworthy that the gene expression patterns of DGCs in the DCKO mice detected on microarray analysis were very similar to those of human primary DGCs other than intestinal ones. Thus, our DCKO mouse model is a powerful tool for investigating the role of gene function in DGC carcinogenesis and for developing a therapeutic strategy. In this study, we established Twist1 expression-positive and -negative DGC cell lines that are derived from the DCKO mice. As a consequence of analysis of the epigenetic alterations, the common regulatory mechanism of *Twist1* was elucidated, and evaluated in both murine and human DGCs in this study.

## Materials and Methods

### Ethics Statement

All *in vivo* procedures were approved by the Animal Care Committee of Tokyo Medical and Dental University (Permission No. 0160073A). As for normal human gastric mucosae samples, written informed consent was obtained from all subjects, and the study was approved by the Ethics Committee of Tokyo Medical and Dental University (No.1115).

### Cell cultures and tissue samples

We previously established an E-cadherin/p53 DCKO (*Atp4b-Cre* ^*+*^; *Cdh1* ^*loxP/loxP*^; *Trp53* ^*loxP/loxP*^) mouse model [19]. From its the cancerous tissues, we established five mouse DGC cell lines and named them MDGCs (Shimada S, unpublished observation). MDGC-1 and MDGC-3 cells were cultured in Dulbecco’s modified Eagle’s medium (DMEM) containing high glucose supplemented with 10% fetal bovine serum, and MDGC-7, MDGC-8 and MDGC-9 were cultured in Ham’s F12 supplemented with 5% horse serum. DNA analysis was performed in order to detect the truncated *Cdh1* and *Trp53* alleles (S1 Fig). Eight human GC cell lines (KATO-III, GCIY, AGS, MKN7, MKN45, MKN74, NUGC4 and HSC60) were also used in this study. MKN7, MKN45, MKN74, GCIY and KATO-III were purchased from RIKEN cell bank (Tsukuba, Japan). NUGC4 and AGS cells were obtained from the Human Science Research Resources Bank (Osaka, Japan) and ATCC (American Type Cell Collection). HSC60 cells were obtained from Dr. Kazuyoshi Yanagihara (National Cancer Research Center, Tokyo, Japan) [24]. KATO-III, MKN7, MKN45, MKN74, NUGC4, AGS and HSC60 cells were cultured in RPMI 1640, and GCIY was cultured in Dulbecco’s modified Eagle’s medium, both of which were supplemented with 10% fetal bovine serum. For de-methylation studies, murine and human GC cells were treated daily with 500nM 5-aza-deoxycytidine (5-aza-dC, Sigma; #A3656) for 72 hours. We treated these GC cells with 100nM trichostatin A (TSA, Wako; #200–11993) for 24 hours or 100nM mithramycin A (Wako; #132–17101) for 24 hours.

Eighteen primary GC tissues and corresponding non-cancerous gastric mucosae were obtained from 15 DCKO mice. As for human stomach tissues, we used three non-cancerous gastric mucosae from GC patients. We also obtained five normal gastric mucosae from *Atp4b-Cre* ^*−*^; *Cdh1* ^*loxP/loxP*^; *Trp53* ^*loxP/loxP*^ mice that were used as negative controls in our previous study [19]. In this study, we called “normal gastric mucosae” if specimens were obtained from control mice, and “non-cancerous gastric mucosae” if specimens were obtained from DCKO mice and human GC patients in this study.

### Reverse transcription (RT)-PCR and quantitative RT-PCR (qRT-PCR)

Total RNA was isolated with an RNeasy Mini kit (Qiagen; #74134) and cDNA was prepared from RNA using a SuperScript III kit (Invitrogen; #18080044) according to the manufacturer’s protocols. End-point RT-PCR carried out with multiple cycle numbers, 28–35 cycles. As an internal control, glyceraldehyde-3-phosphate dehydrogenase (*GAPDH*) was amplified with 21 cycles to ensure cDNA quality and quantity for each RT-PCR. The amplified products were loaded to 2% agarose gels or quantified by quantitative RT-PCR using LightCycler DNA Master SYBR Green I (Roche Diagnostics; #04707516001). The amplification programs for PCR were repeated for 40 cycles for Gapdh and 45cycles for other genes except for Gapdh. PCR products were cloned into the pMD20 T-vector by using a Mighty TA-cloning Kit (TaKaRa BIO INC; #6028), and then sequenced directly. The primer sequences and PCR product sizes are shown in S1 Table.

### Methylation Analysis

We extracted genomic DNA from murine and human GC cell lines by the phenol-chloroform method, and then carried out bisulfite modification and the MSP procedure. Bisulfite treatment of DNA was performed with EZ DNA Methylation-Gold (ZYMO RESEARCH; #D5006), and then methylation-specific PCR (MSP) was conducted. Briefly, the PCR reaction was performed for 35 cycles in a 25 μl mixture comprising bisulfite-modified DNA, 2.5μl of 10x PCR buffer, 1.25 μl of 25mM dNTPs, 25 pmol/l of each primer and 1 U of JumpStart RedTaq polymerase (Sigma; #D0563). The conditions for PCR were as follows: 95°C for 5 minutes; 35 cycles of 95°C for 1minutes, 53°C for 2minutes, and 72°C for 1.5minutes; and a final extension at 72°C for 5 minutes. As for mouse GC tissues, DNA was extracted from formalin-fixed paraffin-embedded (FFPE) tissues. Bisulfite DNA was amplified with flanking PCR primers and then nested MSP was conducted [25, 26]. The primer sequences and PCR product sizes are shown in S2 Table.

### Chromatin immunoprecipitation (ChIP) assay

The ChIP assay was performed using a ChIP-IT Express kit (Active Motif; #53008) according to the manufacturer’s protocol. Immunoprecipitated DNA enrichment was normalized as to the input. The antibodies used in this study were anti-histone H3K4me3 (Active Motif; #39915), anti-H3K9me3 (Active Motif; #39765), anti-H3K27me3 (Active Motif; #39155), anti-H3K36me2 (abcam; #ab9049), anti-Sp1 (abcam; #ab13370), and anti-Histone H3 (Active Motif; #39163) antibodies. Normal rabbit IgG (Cell Signaling Technology; #2729s) was used as a negative control for ChIP. The primer sequences and PCR product sizes are shown in S2 Table.

### Knockdown analysis using small interfering RNAs

siRNA-based knockdown of *Suv39h1* and *Suv39h2* was performed using an electroporation (Neon transfection system, Invitrogen) according to the manufacturer’s instructions. MDGC cells were transfected with 50nM siRNA of Suv39h1 (SASI_Hs02_00335192, Sigma), Suv39h2 (SASI_Hs01_00101226), or negative control siRNA (Mission siRNA Universal Negative Control, Sigma). After 48 hours culturing, cells were harvested for RT-PCR, Western blot analyses, migration, invasion, and proliferation assay.

### Western Blot Analysis

GC cells were lysed with Pierce RIPA buffer (Thermo Scientific; #89900). Proteins were separated on SDS-polyacrylamide gels and then transferred to polyvinylidene fluoride (PVDF) membranes, followed by incubation with antibodies dissolved in Tris-buffered saline (TBS) containing 2% skim milk and 10% Tween 20. The primary antibodies used in this study were mouse monoclonal anti-Twist1 (1:100, Santa Cruz Bio-technology; #sc-81417), rabbit polyclonal anti-Sp1 (1:200, Active Motif; #39058), and mouse monoclonal anti-α-tubulin (1:200, Santa Cruz; #sc-8035) antibodies. The secondary antibodies were alkaline phosphatase-conjugated anti-rabbit IgG (1:3000, Bio-Rad Laboratories; #1706518) and anti-mouse IgG (1:3000, Bio-Rad Laboratories; #1706520). Blots were developed with ImmunoStar AP Substrate (Bio-Rad Laboratories; #1705018).

### Immunohistochemistry

FFPE sections (4 μM) were deparaffinized in xylene, rehydrated in graded ethanol solutions and then Antigen retrieval was conducted in 10 mM citric acid buffer by microwaving. Immunohistochemistry was performed using anti-Twist1 antibodies (Santa Cruz, 1:20) as described previously [19]. Expression of Twist1 was defined as positive nuclear staining in more than 10% of the cells, and the intensity was scored as–(negative), + (weak) and ++ (moderate to strong) by three investigators independently.

## Results

### Twist1 expression in murine and human GC cell lines

**Fig 1:**
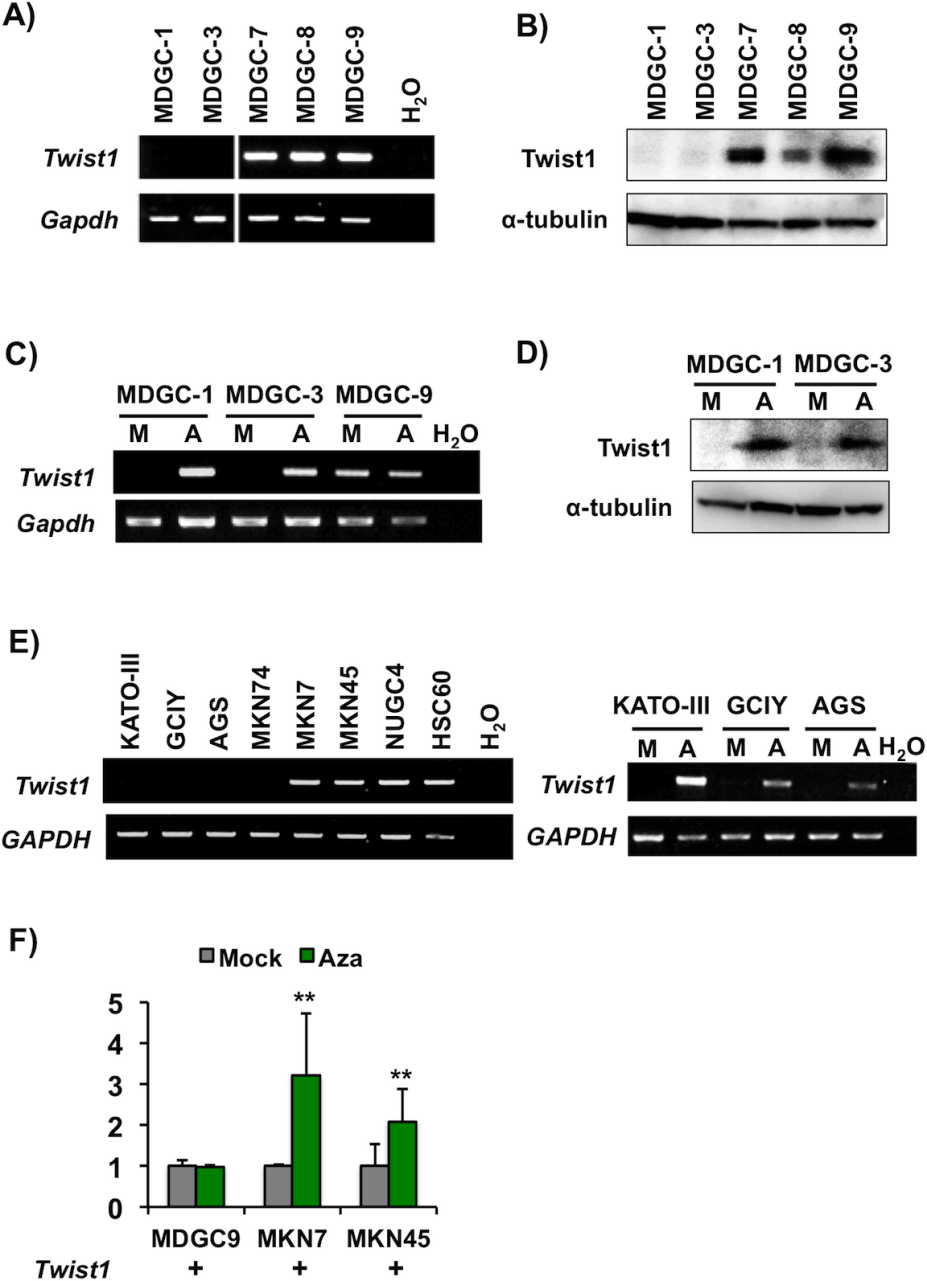
Twist1 expression in murine and human GC cell lines. (A) RT-PCR analysis of *Twist1* mRNA expression in five mouse GC cell lines MDGC-1, MDGC-3, MDGC-7, MDGC-8 and MDGC-9) from DCKO mice. Mouse *Gapdh* was used as an internal control. (B) Expression of Twist1 protein in MDGC cells by Western blot. α-tubulin was used as an internal control. (C and D) Effects of a DNA demethylation agent in MDGC cells. After treatment with 5-aza-dC, *Twist1* expression was up-regulated at the mRNA (C) and protein levels (D) in Twist1 expression-negative MDGC-1 and MDGC-3 cells, but not in Twist1 expression-positive MDGC-9 cells. M, mock; A, 5-aza-dC. (E) RT-PCR analysis of *Twist1* mRNA expression in human GC cell lines (KATO-III, GCIY, AGS, MKN74, MKN7, MKN45, NUGC4 and HSC60) (left). *Twist1* expression was up-regulated at the mRNA level in KATO-III and GCIY cells after 5-aza-dC treatment (right). Human *GAPDH* was used as an internal control. (F) qRT-PCR analysis of *Twist1* mRNA expression in MDGC-9, MKN7 and MKN45 cells with and without 5-aza-dC treatment (**P<0.01).

From DGC tissues of DCKO mice, *Twist1* transcription-positive cell lines (MDGC-7, MDGC-8 and MDGC-9) and negative cell lines (MDGC-1 and MDGC-3) were established (Fig 1A and S2A Fig), and the expression of Twist1 protein was confirmed in each cell line (Fig 1B). Twist1 expression was enhanced at the mRNA and protein levels in Twist1 expression-negative MDGC cells after treatment with DNA demethylation agent 5-aza-dC (Fig 1C and 1D), while its expression was not changed after treatment with histone deacetylase inhibitor TSA (S2B Fig). In the eight human GC cell lines examined, *Twist1* expression was downregulated in four cell lines (Fig 1E, left), three (KATO-III, GCIY and AGS) of which demonstrated the re-activation of *Twist1* expression after treatment with 5-aza-dC by RT-PCR (example, Fig 1E, right). As for *Twist1* expression-positive GC cell lines, after 5-aza-dC treatment, 2.1- and 3.2-fold up-regulation of *Twist1* was detected in MKN45 and MKN7, respectively, by qRT-PCR, whereas Twist1 expression was not changed in MDGC-9 cells (Fig 1F).

### CGI methylation and expression of *Twist1* in GC cell lines

**Fig 2:**
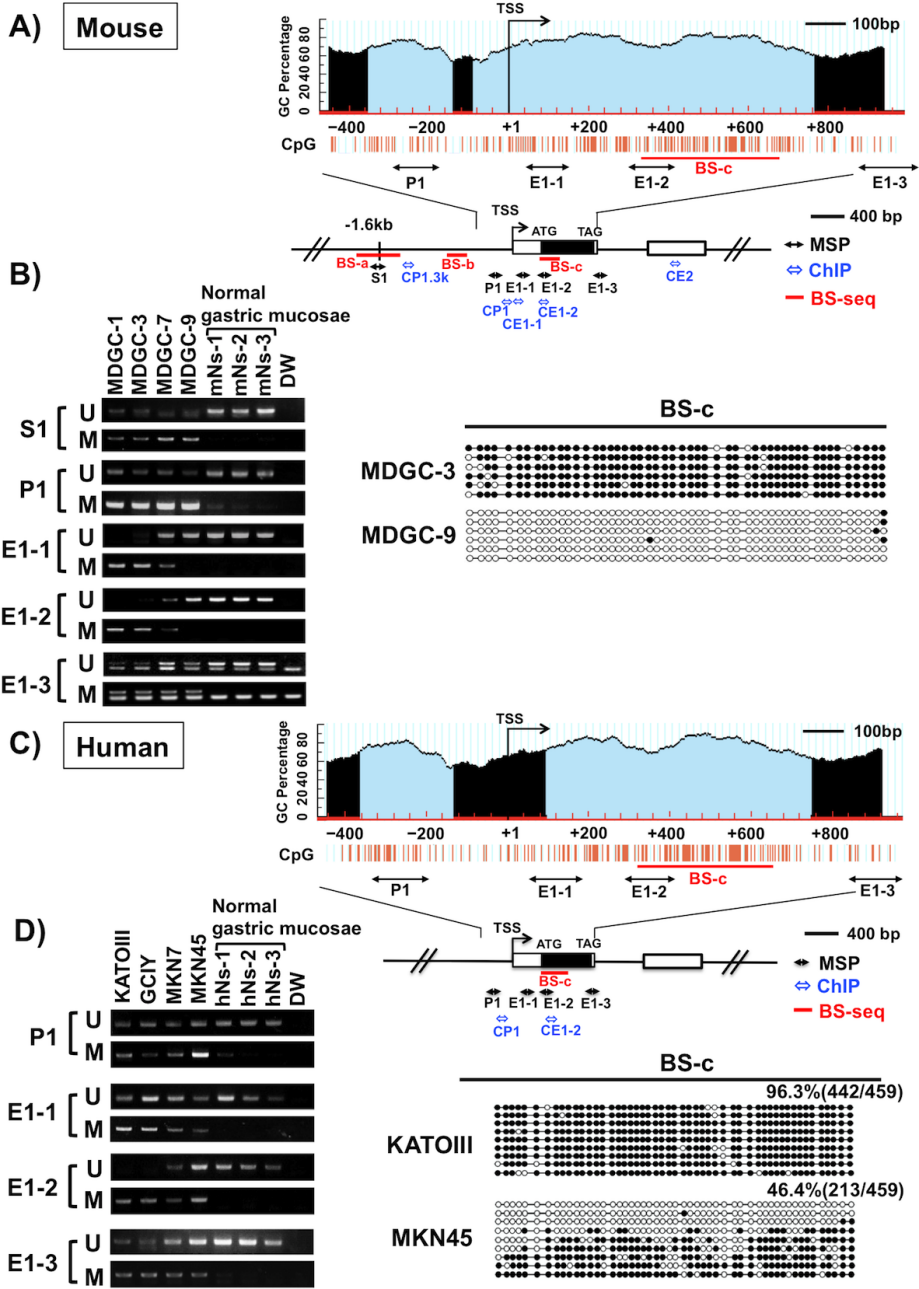
*Twist1* methylation status in murine and human GC cell lines. (A) Schematic representation of the genomic structure and the predicted CGI with MethPrimer (http://www.urogene.org/methprimer/) of the mouse *Twist1* gene. Two CGIs (thin blue regions, left, -358 to -149; right, -96 to +759) were observed at the region from the promoter to entire exon 1 (Obs/Exp = 0.8, %GC> 50%). The bent arrow indicates the transcription start site (TSS). Individual CpG sites are indicated as orange vertical lines. The boxes denote the exon. The black double-headed arrows indicate the regions (S1, P1, E1-1, E1-2 and E1-3) examined by methylation specific PCR (MSP). The blue double-headed arrows indicate the regions examined by ChIP assay. The red bars indicate the regions examined by bisulfite sequencing (BS). (B) MSP analysis of MDGC cell lines and three normal gastric mucosae from *Atp4b-Cre* ^*-*^; *Cdh1* ^*loxP/loxP*^; *Trp53* ^*loxP/loxP*^ mice in the five regions (left). U: unmethylated alleles; M: methylated alleles. BS of the region from +322 to +670 (BS-c) in MDGC-3 and MDGC-9 (right). BS-a and BS-b are shown in the S3 Fig. The MSP region of E1-2 and BS-c correspond to *Twist1* expression. Black circles represent the methylated CpG site; white circles represent the unmethylated CpG sites. (C) Schematic representation of the genomic structure and the predicted CGI with MethPrimer of the human *Twist1* gene. Two CGIs (left, -369 to -138; right, +91 to +742) were observed (Obs/Exp = 0.8, %GC> 50%). (D) MSP and BS analyses of human GC cell lines and three non-cancerous gastric mucosae from GC patients. We assessed four regions (left, P1, E1-1, E1-2 and E1-3) and exon 1 (right, +315 to +651, BS-c) in the human *Twist 1* gene, which are located analogously to mouse MSP and BS regions. Dense methylation was detected in KATO-III cells lacking *Twist1* expression, while MKN45 cells expressing *Twist1* exhibited both methylated and unmethylated alleles.

*Twist1* has a dense CGI region from the promoter to the entire exon 1, this region being highly conserved among vertebrates including mouse and human (S3 Fig). To clarify the CGI in mouse and human *Twist1*, we performed computational analysis using UCSC Genome Browser (http://genome.ucsc.edu/index.html) and MethPrimer (http://www.urogene.org/methprimer/) that is widely used as identification of CGI and MSP primers [27]. We analyzed approximately 1.4 kb region including the promoter and entire exon 1. UCSC Genome Browser on mouse and human analysis exhibited one CGI in the region examined (data not shown). However, putative two CGI sites were detected in the region in both mouse and human when we used the criteria of CGI with GC content of 50% and observed/expected CpG ratio of 0.8 by MethPrimer (Fig 2A and 2C). These two putative CGI regions were located at the promoter and exon 1. Since the methylation at the promoter region has been reported in various human cancers [12, 14], we initially examined the methylation status at the region in murine and human GC cell lines by MSP. However, the methylation status at the promoter region of *Twist1* did not correspond to its expression in any GC cell lines examined (region P1, Fig 2A). To determine the critically methylated regions associated with Twist1 expression in MDGC cells, we further performed MSP at the CGI region in exon 1 (E1-1, E1-2 and E1-3), and the predicted CpG shore site located approximately 1.6 kb from TSS (transcript start site, S1) (Fig 2A). As a result, CGI methylation at the region (E1-1 and E1-2) around the TSS in *Twist1* exon 1 was found to correspond to expression of the gene in MDGC cells, though there was no correlation between them at the predicted shore region (S1) and the end of the exon 1 region (E1-3) (Fig 2B, left) in MDGC cells. No methylation was detected at any CpG regions in three normal gastric mucosae without Twist1 expression from *Atp4b-Cre* ^*-*^; *Cdh1* ^*loxP/loxP*^; *Trp53* ^*loxP/loxP*^ mice (Fig 2B). In human gastric mucosae, no *Twist1* methylation was also found in three non-cancerous gastric mucosae from GC patients (Fig 2D), one of which showed very weak *Twist1* expression (data not shown). We also examined additional four non-cancerous gastric mucosae from GC patients, while no methylation was detected at E1-2 region in any cases (data not shown). Bisulfite sequencing (BS) demonstrated that the CGI region in exon 1 showed dense methylation in Twist1 expression-negative MDGC-3 cells but not in Twist1-positive MDGC-9 cells (BS-c, Fig 2A and 2B, right), while there was no difference in the methylation patterns between them at the CpG shore and promoter regions (BS-a and BS-b, S4A Fig). Among the human GC cell lines, KATO-III and GCIY without *Twist1* expression showed dense CGI methylation at the region of exon 1 (E1-2) which is consistent with ones of mouse *Twist1* (Fig 2C and 2D, left). Both methylation and unmethylation in the E1-2 region were detected in MKN7 and MKN45 cells by MSP and bisulfite sequencing (Fig 2D), which are basally expressed Twist1 but their expression levels were increased after 5-aza-dC treatment (Fig 1F). Thus, our data indicate that methylation in the exon 1 region of *Twist1* detected in this study is correlated to its expression.

### *Twist1* methylation and expression in DCKO mouse GC tissue samples

**Fig 3:**
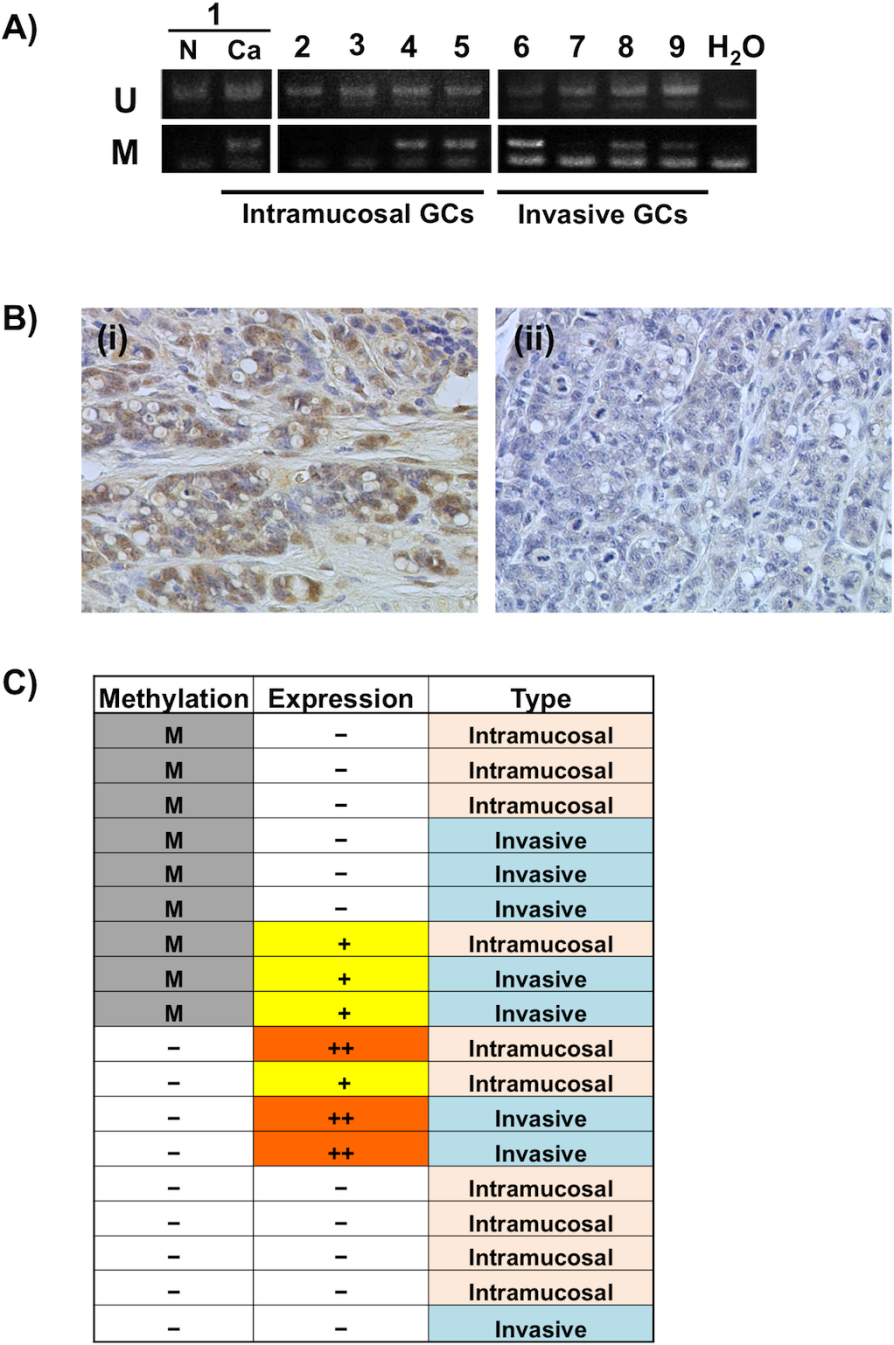
The expression and methylation status of *Twist1* during cancer progression. (A) Representative results of nested MSP analysis of *Twist1* in DGCs from DCKO mice and a corresponding non-cancerous gastric mucosa (lane N). The regions (E1-2) analyzed are shown in Fig 2A. U: unmethylated allele; M: methylated allele. (B) Representative immunohistochemical staining of Twist1 protein in DGC tissues from DCKO mice. (i) Strong nuclear staining of Twist1 protein in a GC without methylation. (ii) Negative Twist1 protein expression in a GC with methylation. Original magnification, x400. (C) Summary of *Twist1* methylation and expression in primary DGCs. *Twist1* was methylated in 9 of 18 GCs, as indicated by M. The intensity of Twist1 staining was scored as–(negative), + (weak) and ++ (moderate to strong). Mouse GCs were divided into two groups, intramucosal and invasive GCs according to the criteria of human GC established by the Japanese Gastric Cancer Association [53].

We examined the methylation status of *Twist1* using 18 primary GC tissues from 15 DCKO mice. Because GC regions were very small and their DNA from FFPE samples showed low concentrations, we performed nested MSP [25, 26]. *Twist1* was significantly methylated (9/18, 50%) in primary GCs compared to corresponding non-cancerous tissues (1/10, 10%), being statistically significant difference (P<0.05, Table 1). Representative data by MSP are shown in Fig 3A. Among them, 4 of 10 (40%) cases were intramucosal and 5 of 8 (62.5%) were invasive GCs (Table 1). In this study, *Twist1* methylation-positive cases demonstrated unmethylated MSP bands as well, which due to the presence of normal stromal/fibroblast and inflammatory cells in cancer tissue sections, although we could not deny the possibility of partial methylation like MKN45 and MKN7 and tumor heterogeneity.

**Table 1:** Frequency of *Twist1* methylation in DGC from DCKO mice.

Sample type		Total	Methylation frequency(%)		IHC expression^5)^			
					++	+	—	frequency(%)
Normal gastric mucosa ^1)^		3	0	(0)	ND	ND	ND	ND
Non-cancerous gastric mucosa bearing GCs ^2)^		10	1	(10)^6)^	0	0	10	(0) ^7)^
GCs		18	9	(50)^6)^	3	4	11	(38.9)^7)^
	Intramucosal GCs ^3)^	10	4	(40)	1	2	7	(30)
	Invasive GCs ^4)^	8	5	(62.5)	2	2	4	(50)

1) Gastric mucosa from control mice (*Atp4b-Cre* ^*−*^; *Cdh1* ^*loxP/loxP*^; *Trp53* ^*loxP/loxP*^ mice)

2) Gastric mucosa from DCKO mice bearing GCs

3) GC confined to the mucosa or submucosa

4) GC invades the muscularis propria, subserosa or serosa

5) The intensity of nuclear staining was scored as + (weak), and ++ (moderate to strong) in more than 10% of the tumor cells. The frequencies of Twist1 positive (++, +) are calculated.

6) p < 0.05 Fisher's exact test

7) p < 0.05 Fisher's exact test

Twist1 protein expression was detected in 7 of 18 (38.9%) GCs compared to corresponding non-cancerous gastric mucosae by immunohistochemistry. Representative pictures are shown in Fig 3B. We further analyzed the relationship between *Twist1* expression and methylation levels in these cases. Among the 18 primary GC examined, nine cases showed correlation between *Twist1* methylation and expression (Fig 3C). Three of four cases with Twist1 weak expression exhibited its methylation, possibly that low expression of Twist1 may be caused by its methylation. However, remaining five cases exhibited no methylation and expression (Table 1).

### Histone methylation is related to the regulation of *Twist1* expression

**Fig 4:**
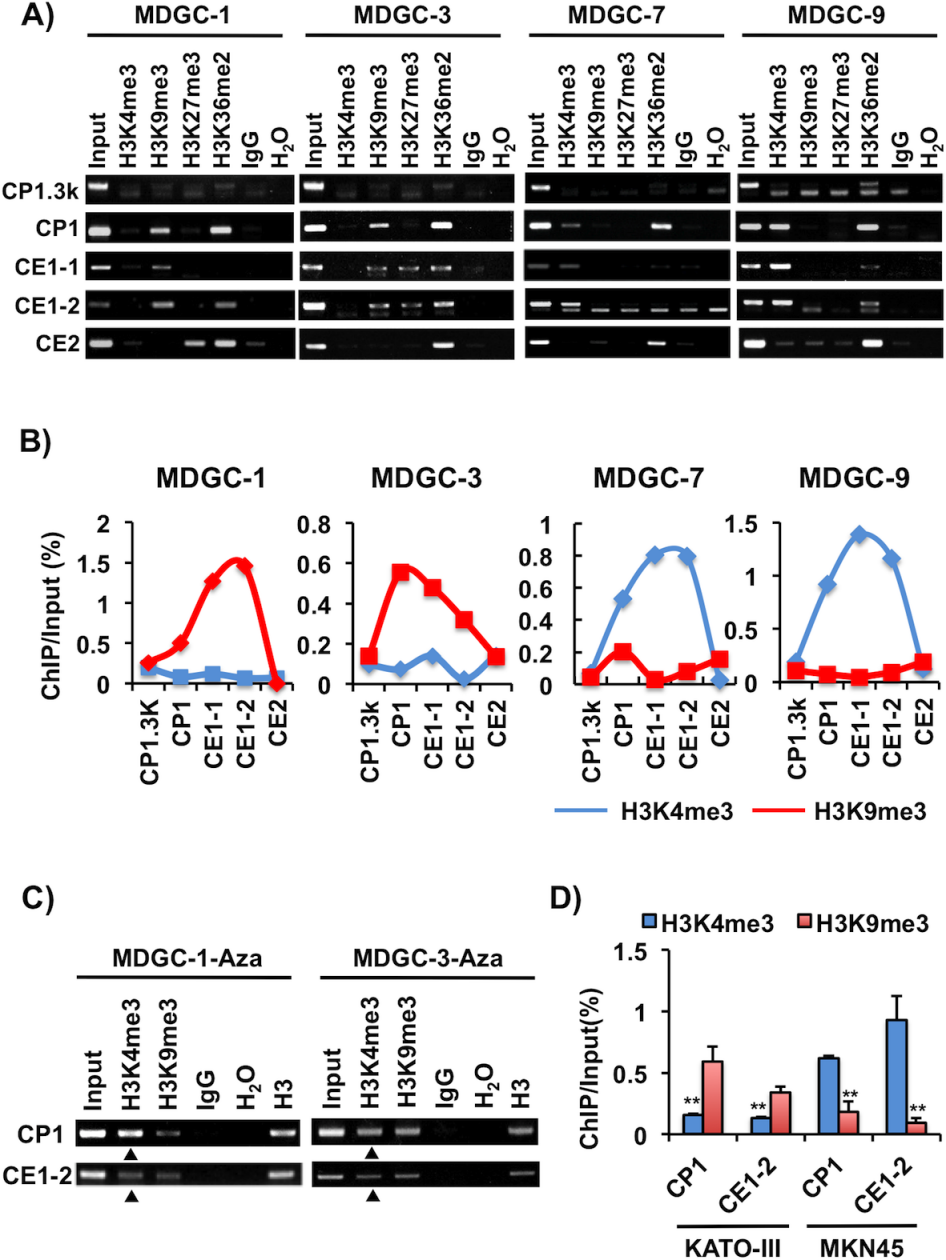
Differences of histone methylation status depending on Twist1 expression in murine and human GC cells. (A) Histone methylation status in Twist1 expression-positive cells (MDGC-7 and MDGC-9) and -negative cells (MDGC-1 and MDGC-3). ChIP assay was conducted using H3K4me3, H3K9me3, H3K27me3 and H3K36me2 antibodies. The five ChIP regions (CP1.3k, CP1, CE1-1, CE1-2 and CE2) analyzed are shown in Fig 2A. Input DNA samples were used as internal controls. (B) Semi-quantitative ChIP analyses of histone H3K4me3 and H3K9me3 enrichment. ChIP intensities were analyzed using Image J 1.47v software, and then ChIP/Input was calculated. Active histone mark H3K4me3 was enriched in Twist1 expression-positive cells, but inactive histone mark H3K9me3 was enriched in Twist1 expression-negative cells. (C) The relationship between histone and CGI methylation in MDGC cells. MDGC-1 and MDGC-3 cells were treated with 5-aza-dC, and the histone methylation status of their H3K4me3 and H3K9me3 was examined by ChIP assay. The H3K4me3 levels were increased in these 5-aza-dC-treated cells compared in untreated controls (Fig 4A), as shown by triangles. (D) Semi-quantitative ChIP analysis of H3K4me and H3K9me3 in human GC cells. The regions (CP1, CE1-2) analyzed are shown in Fig 2C. Similar to the data for mouse *Twist1* (Fig 4B), the H3K4me3 and H3K9me3 levels were enriched in MKN45 and KATO-III cells, respectively. The average (column) ± S.D (bar) for three independent agarose gel electrophorese in different experiments is indicated (**P<0.01).

We next investigated whether or not the lysine methylation level of histone H3 contributes to *Twist1* expression. Chromatin immunoprecipitation (ChIP) assays were performed on the predicted CpG shore (CP1.3k), promoter (CP1), and exons 1 (CE1-1 and CE1-2) and 2 (CE2) regions (Fig 2A). At the CP1 and CE1-2 regions, active histone mark H3K4me3 was enriched in Twist1 expression-positive cells (MDGC-7 and MDGC-9), but inactive histone mark H3K9me3 was enriched in Twist1 expression-negative cells (MDGC-1 and MDGC-3) (Fig 4A and 4B). ChIP assays exhibited both H3K4me3 and H3K9me3 signals in MDGC-1 and MDGC-3 cells with 5-aza-dC treatment, indicating the elevated H3K4me3 levels in these cells (Fig 4C). On the contrary, the H3K27me3 and H3K36me2 levels were not correlated with Twist1 expression in any MDGC cells examined in this study. As for human GC cell lines, a similar region located from the promoter to exon 1 showed H3K4me3 enrichment in *Twist1* expression-positive MKN45 cells, and H3K9me3 in *Twist1* expression-negative KATO-III cells (Figs 2C and 4D).

### *Suv39h1* and *Suv39h2*, a histone methyltransferase, regulates H3K9me3

**Fig 5:**
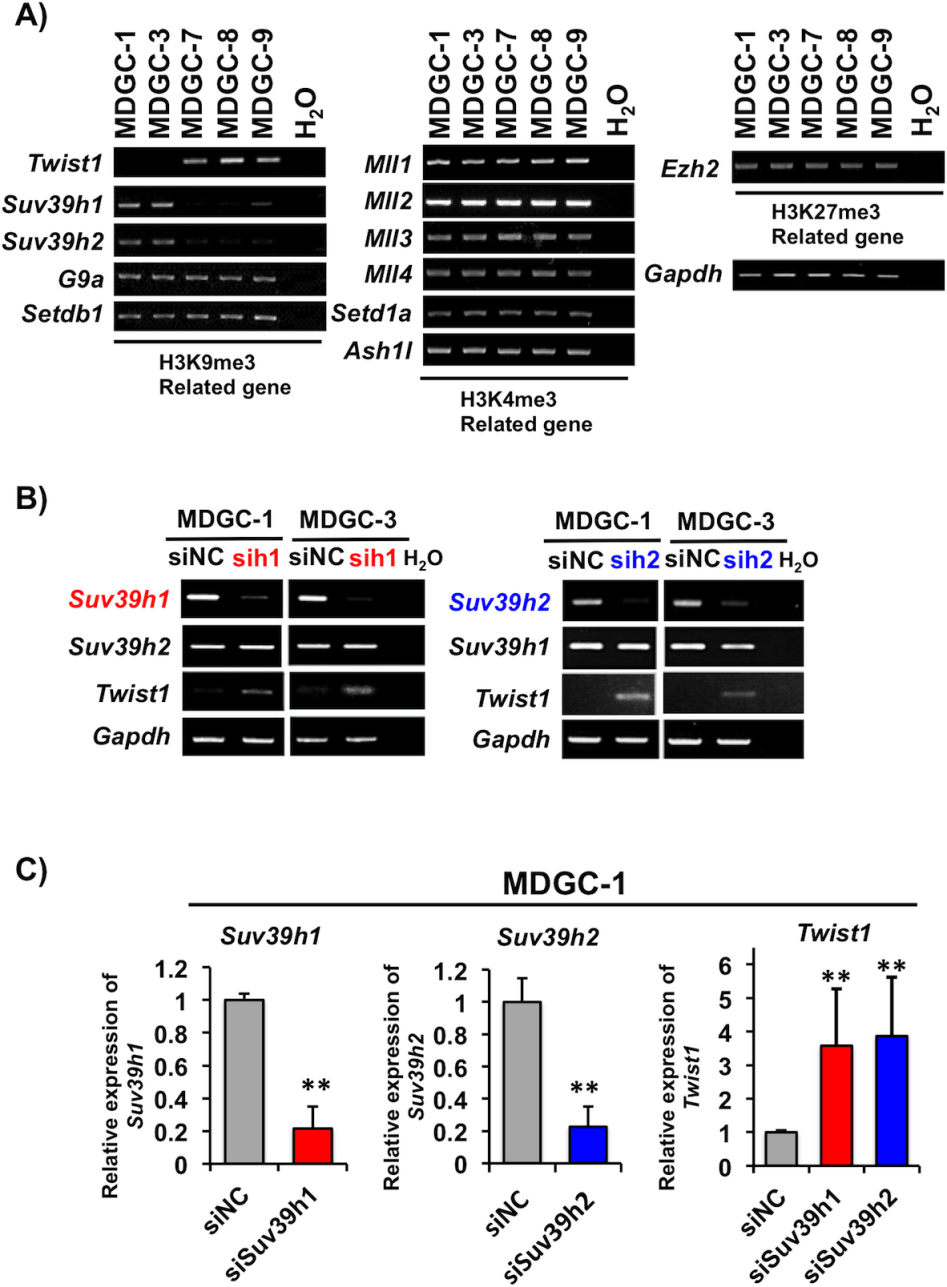
Expression of histone methyltransferase *Suv39h1* and *Suv39h2* in MDGC cells. (A) Expression of histone methyltransferases for H3K4 (*Mll1*, *Mll2*, *Mll3*, *Mll4*, *Setd1a and Ash1l*), H3K9 (*Suv39h1*, *Suv39h2*, *G9a and Setdb1*), and H3K27 (*Ezh2*) was determined by RT-PCR. The expression levels of *Suv39H1* and *Suv39H2* were inversely correlated with *Twist1* expression. *Gapdh* was used as an internal control. (B and C) Effect of *Suv39h1* or *Suv39h2* knockdown on *Twist1* expression in MDGC cells. RT-PCR was performed on MDGC-1 and MDGC-3 cells with *Twist1* expression after transfection of si*Suv39h1* (sih1), si*Suv39h2* (sih2) or negative control (siNC) siRNA. (C) The *Twist1*, *Suv39h1* and *Suv39h2* mRNA levels in MDGC-1 cells were further confirmed by qRT-PCR. The columns and bars indicate averages and S.D., respectively. **P<0.01.

There are a lot of histone methyltransferases associated with H3K4, H3K9 and H3K27 [28]. Therefore, to determine which histone modification enzymes are responsible for the H3K4me3 or H3K9me3 enrichment at the *Twist1* exon 1 region in GC cells, we examined ten H3K4me3 (*Mll1*, *Mll2*, *Mll3*, *Mll4*, *Setd1a*, *Setd1b*, *Ash1*, *Ash1l*, *Smyd3* and *Meisetz*), seven H3K9me3 (*Suv39h1*, *Suv39h2*, *G9a*, *Setdb1*, *Clld8*, *Glp* and *Riz1*), and one H3K27me3 (*Ezh2*) related genes. Representative data are shown in Fig 5A. Among them, the expression levels of *Suv39h1* and *Suv39h2* were inversely correlated with *Twist1* expression. However, the expression levels of remaining 16 histone methylatasferase genes examined were not associated with *Twist1* expression in MDGC cells. We performed siRNA-based knockdown of *Suv39h1* or *Suv39h2* in the respective expression-positive MDGC-1 and MDGC-3 cells by electroporation (Fig 5B). Knockdown of *Suv39h1* or *Suv39h2* in these cell lines led to an increase of *Twist1* expression observable on RT-PCR (Fig 5B) and qRT-PCR (MDGC-1, Fig 5C).

### Association of Sp1 with the transcriptional regulation of *Twist1*

**Fig 6:**
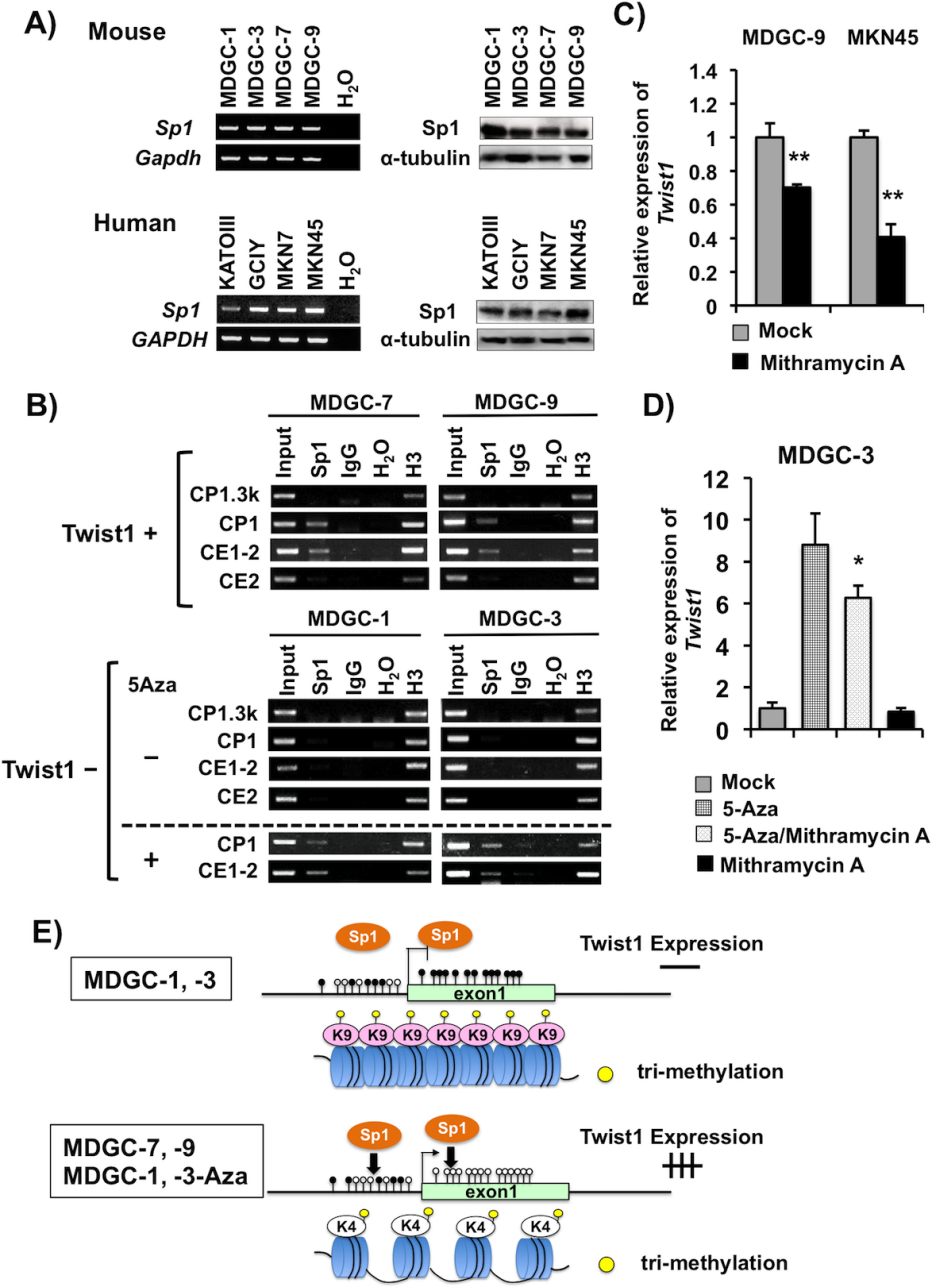
*Twist1* expression elevated by binding of Sp1 transcription factor. (A, B) RT-PCR (left) and Western blot (right) analyses of *Sp1* mRNA and protein in MDGC cells and human GC cells. *Gapdh* and α-tubulin were used as internal controls for RT-PCR and Western blot, respectively. (B) ChIP assay showing the difference in Sp1 binding level between Twist1 expression-positive and -negative cells. Sp1 strongly bound to the CP1 and CE1-2 regions in Twist1 expression-positive cells (MDGC-7, MDGC-9). After 5-aza-dC treatment, Sp1 binding increased in Twist1 expression-negative cells (MDGC-1, MDGC-3). The regions analyzed are shown in Fig 2A. (C) Inhibition of *Twist1* mRNA expression in murine and human GC cells after treatment with mithramycin A, a Sp1 binding inhibitor. (D) Alterations of *Twist1* expression in MDGC-3 cells after treatment with 5-aza-dC, mithramycin A, and the combination. *Twist1* expression was enhanced in MDGC-3 after treatment with 5-aza-dC but not mithramycin A. The *Twist1* expression enhancement by 5-aza-dC was significantly inhibited by mithramycin A (*P<0.05). (E) Schematic representation of the transcriptional regulatory mechanisms of *Twist1* in association with the epigenetic machinery and transcription factors. It is possible that *Twist1* transcriptional activation is regulated through potential cooperation of DNA methylation, histone modification in complex with Sp1 binding to CpG-rich regions in exon 1.

The consensus 5'-(G/T)GGGCGG(G/A)(G/A)(C/T)-3' sequence is known as the binding motif of Sp1 transcription factor, and a relationship between CGI methylation and Sp1 binding in the promoter has been reported [29, 30]. Multiple Sp1 binding sites were predicted in the CpG-rich region within *Twist1* exon 1 with TFBIND (http://tfbind.hgc.jp) and JASPAR (http://jaspar.binf.ku.dk). The role of Sp1 in the transcription of *Twist1* was then examined with reference to epigenetic modification of the gene. We detected expression of Sp1 mRNA and protein in all murine and human GC cells examined, even in Twist1 expression-negative MDGC-1, MDGC-3, KATOIII and GCIY cells (Fig 6A).

To determine whether or not Sp1 binds to the regions of the *Twist1* promoter and exon 1 in GC cells, we performed ChIP assay of Sp1 using MDGC cells (Fig 2A). Sp1 bound to the regions of the *Twist1* promoter (CP1) and exon 1 (CE1-2) in Twist1 expression-positive cell lines (MDGC-7 and MDGC-9) (Fig 6B). In contrast, Twist1 expression-negative cell lines (MDGC-1 and MDGC-3) showed undetectable levels of Sp1 binding signals at the CP1 and CE1-2 regions. Sp1 did not bind to any regions of the predicted CpG shore (CP1.3k) and exon 2 (CE2) regions in these Twist1 expression-positive and -negative MDGC cells. Demethylation with 5-aza-dC increased Sp1 binding at the *Twist1* promoter and exon 1 regions in the Twist1 expression-negative MDGC-1 and MDGC-3 cells (Fig 6B). Mithramycin A is known to interfere with Sp1 binding to CpG-rich regulatory sequences [31, 32]. It was noted that *Twist1* expression was down-regulated after treatment with mithramycin A in its expression-positive GC cells (MDGC-7, MDGC-9, MKN7 and MKN45 cells) (Fig 6C and S6 Fig). Mithramycin A did not show any effect on Sp1 expression in these cells (data not shown). Up-regulation of *Twist1* by 5-aza-dC was decreased by mithramycin A treatment in MDGC-3 cells (P = 0.04, Fig 6D).

## Discussion

The mechanisms underlying epigenetic regulation of oncogene are poorly understood. The relationship between methylation status at the *Twist1* promoter regions and expression of *Twist1* is controversial, and there are almost none of reports on altered histone modification in *Twist1* expression. Thus, it remains uncertain whether or not epigenetic changes are related to *Twist1* activation in cancer cells. As new findings in this study, we elucidated the *Twist1* regulatory mechanism underlying fundamental epigenetic machinery that DNA methylation, histone modification and Sp1 act in concert with Twist1 expression in GC cells.

It is widely known that DNA methylation at the CGI promoter region is critical for gene silencing, and aberrant CGI methylation results in loss of expression of various TSGs [10]. Twist1 was re-activated in both murine and human GC cells with 5-aza-dC treatment in this study, indicating that transcriptional activation of Twist1 is also modulated by DNA methylation. CGI methylation of the *Twist1* promoter region has been found in various cancers such as gastric, breast, colorectal and lung ones [11, 12, 14], most of which showed no correlation between *Twist1* methylation and expression. We observed that CGI methylation in *Twist1* exon 1 other than in the promoter region corresponded to its gene expression in mouse GC cells. As for human GC cell lines, *Twist1* expression-negative cell lines were fully methylated, but its expression-positive cell lines exhibited both methylation and unmethylation of *Twist1*, all of which up-regulated *Twist1* expression after 5-aza-dC treatment (Fig 1F). These data indicate that MKN45 and MKN7 cells showed partially methylation of *Twist1*, and half levels of *Twist1* expression were basally detected in these cells, which were expressed from unmethylated DNA allele. Recent reports indicated that CpG shore methylation is related to gene expression [7, 33]. However, the methylation status at the predicted CpG shore site region was not correlated with *Twist1* expression. Our data indicate that *Twist1* is epigenetically silenced by DNA methylation in murine and human GC cell lines.

According to the computational analysis of mouse and human CGIs from the promoter to entire coding region in the *Twist1* gene, two putative CGI regions were located at the promoter and exon 1 by MethPrimer analysis. Here we observed that CGI methylation at the exon 1, rather than its promoter region, was correlated with *Twist1* expression in GC cells. Such distinct methylation patterns associated with gene silencing have been reported in several genes [26, 34, 35]. For example, *SOX2* and *AP2α* exhibited putative two CGIs at the promoter and exon 1, and methylation at the exon 1 region has shown to be significantly correlated to its gene expression in GCs and breast cancers [34, 35]. It was reported that CGI methylation around the translational start site in the retinoic acid receptor beta (RAR-ß) gene dramatically corresponded to its expression in murine lung cancers compared to methylation at the promoter region located in same CGI [26]. Thus, our data suggest that CGI methylation at the exon 1 region in *Twist1* is important to its gene silencing in GC cells. However, it is unknown the mechanism of this discrepant methylation between the promoter and exon 1 regions. Several transcription factor binding sites, such as Sp1 elements, and histone modification have been reported to protect CGI methylation [36, 37]. It is important to examine these elements and epigenetic machinery in the CGI regions of *Twist1* further.

In primary GCs from DCKO mice, 50% of GCs exhibited *Twist1* methylation by nested MSP. Our DCKO mice show parietal cell-related atypical foci in stomach, and intramucosal DGCs composed of poorly differentiated cells and signet ring cells often detected at 6 months. Therefore, we have previously proposed that our DGCs in DCKO mice developed through the parietal cell lineage [19]. *Twist1* was methylated in 40% of intramucosal cancers, while the frequency of methylation in non-cancerous gastric mucosae including atypical foci was rare (10%), indicating that *Twist1* methylation is an early event in DGC carcinogenesis in our mouse model. Among the 18 primary GCs examined, five cases were both *Twist1* methylation- and expression-negative. Because non-cancerous gastric mucosae showed undetectable levels of *Twist1* expression and methylation, these GCs may not link to *Twist1* alterations. Except these cases, it is possible that *Twist1* methylation is associated with loss or low expression in primary GCs from DCKO mice.

In addition to DNA methylation, histone modification is also a pivotal key for the control of gene expression. We found that H3K4me3 was enriched in *Twist1* expression-positive cells (MDGC-7, MDGC-9 and MKN45), but H3K9me3 was enriched in *Twist1* expression-negative cells (MDGC-1, MDGC-3 and KATO-III) at the region from the promoter to exon 1 of the *Twist1* gene. Our findings are consistent with the previous observation that active transcription is involved in H3K4me2/3, and inactive transcription is associated with H3K9me2/3 [38]. In spite of the fact that H3K36me2 promoted by MMSET/WHSC1 has been linked to TWIST1 expression, no correlation between H3K36me2 and *Twist1*-expression was observed in our study [39]. Thus, our data indicate that histone marks of H3K4 and H3K9 are implicated in regulation of *Twist1* in GCs.

There are many histone methyltransferases that are specific for the lysine residues in histone proteins; *Mll1/2/3/4*, *Setd1a* and *Ash1l* for H3K4me3, *Suv39h1/h2*, *G9a* and *Setdb1* for H3K9me3, and *Ezh2* for H3K27me3 [28]. In this study, we observed that the expression levels of *Suv39h1* and *Suv39h2* were inversely correlated with *Twist1* expression, and knockdown of either *Suv39h1* or *Suv39h2* increased *Twist1* expression. Overexpression of SUV39h1 and SUV39H2 has been reported in various cancers [40, 41]. These reports also showed that undetectable or low expression levels of these two genes in normal tissues and corresponding non-cancerous tissues. Similarly, no or low expression levels of *Suv39h1* and *Suv39h2* were observed in normal gastric mucosae from GCs from *Atp4b-Cre* ^*−*^; *Cdh1* ^*loxP/loxP*^; *Trp53* ^*loxP/loxP*^ mice in our study (S5 Fig), suggesting that MDGC-1 and MDGC-3 without Twist1 expression may be related to acquisition of these two genes regulation. Suv39h1 and Suv39h2 have been reported to be associated with gene regulation by repressing H3K9me3 and stability of heterochromatin [42–45]. Chromatin is structurally divided into euchromatin and heterochromatin, which are responsible for gene silencing in collaboration with DNA methylation and histone modification [7, 8, 38]. Histone H3K4me3 and H3K9me3 are highly enriched in euchromatin and heterochromatin [7, 8], respectively. Our data suggested that the region from promoter to exon 1 in Twist1 expression-negative cell lines was in the heterochromatin state by Suv39h1 and Suv39h2. In Twist1 expression-positive cell lines, on the other hand, the region was expected to be in the euchromatin state through H3K4me3 enrichment and CGI unmethylation. It might be worthy of evaluation in further studies to identify the H3K4me3-related genes.

In general, Sp1 binds to the consensus GC-box DNA recognition elements and CCT repeats at the gene promoter region [46, 47]. It was recently reported that Sp1/KLF1 binding to the elements in exon 1 with a CpG-rich sequence is a critical event in transcriptional regulation of *TMSG1* (tumor metastasis suppressor gene-1) and *PAX6* (paired box protein-6) [48, 49]. We also found that Sp1 bound to *Twist1* exon 1 in Twist1 expression-positive GC cells but not in negative ones. This CGI region in exon 1 of the *Twist1* gene is evolutionarily conserved in vertebrates and contains several Sp1 binding motifs (S3 Fig). Hence, Sp1 binding to the CGI in *Twist1* exon 1 may play a role in its transcriptional regulation. It is known that Sp1 binding is inhibited by CGI methylation of its binding sequences in several genes, such as *FOXF2* and *p21* [29, 50]. We observed that CGI methylation in exon 1 of the *Twist1* gene was inversely correlated with Sp1 binding, and the administration with 5-aza-dC enhanced the Sp1 binding capacity in both of the promoter and exon 1 regions in Twist1 expression-negative GC cells. These data further support that Sp1 binding may be greatly affected by DNA methylation.

The molecular mechanism underlying oncogenic activation through epigenetic alterations is uncertain. In our study, *Twist1* was unmethylated, and *Suv39h1* and *Suv39h2* expression were undetectable or very low in normal gastric mucosae. Nevertheless, normal gastric mucosae did not show any Twist1 expression, leading us that additional mechanisms, such as transcription factors, may be necessary to up-regulation of Twist1 in gastric carcinogenesis. Sp1 is overexpressed in various cancers including GC, but not in normal tissues [51, 52]. Sp1 was expressed in all MDGC and human GC cell lines, but Sp1 bound the umnethylated alleles of *Twist1* in this study. Therefore, it is tempting to speculate that both Sp1 overexpression and its binding to unmethylated *Twist1* might play a part in carcinogenesis. In MDGC cells, it is possible that unmethylated CGI of exon 1 may loosen the chromatin structure of the region from its exon1 to promoter through H3K4me3. Sp1 may bind to the region, resulting in upregulation of *Twist1* expression. On the contrary, CGI methylation and the subsequent condensed chromatin state through H3K9me3 at the region may interfere with the binding of the Sp1, resulting in inhibition of *Twist1* expression (Fig 6E).

In summary, we found that DNA and histone methylation at the CpG-rich region in *Twist1* exon 1 is strongly correlated with expression of the gene. We also revealed the possibility of transcriptional regulatory mechanisms via cooperation with epigenetic machinery and Sp1 binding in GCs, which is common in murine and human GC cells. Further studies are required to reveal the clinical impact of the complex regulation of Twist1 expression in tumorigenesis.

## Supporting Information

S1 Fig DNA analysis to confirm the truncated *Cdh1* and *Trp53* alleles in MDGC cells, transplanted DGC and normal other tissues. (A) Schematic representation of the genomic structures of *Trp53* sequences and their truncated regions. The filled boxes denote the exon. The blue and pink arrows indicate the regions examined by RT-PCR. The numbers indicate exon numbers. (B) Genomic PCR analysis of truncated *Cdh1* and *Trp53* alleles in MDGC cells. DNA was amplified by PCR using primer, shown in S1 Table. DGC tumors from DCKO mice were transplanted to nude mice. Genomic DNA extracted from the transplanted DGC and DCKO mouse tails was used as positive controls for the truncated and not-truncated alleles of these two genes, respectively. *Atp4b-Cre* was used as an internal control.
(TIF)

S2 Fig *Twist1* mRNA expression in MDGC cells. (A) RT-PCR analysis of The *Twist1* mRNA expression in GC cells. *Twist1* expression was not changed when the cells cultured in different mediums. D, Dulbecco’s modified Eagle’s medium containing high glucose supplemented with 10% fetal bovine serum; H, Ham’s F12 supplemented with 5% horse serum. (B) The *Twist1* mRNA levels did not change after treatment with TSA in Twist1 expression-positive (MDGC-9) and -negative (MDGC-1, MDGC-3) cells. M, mock; T, TSA.
(TIF)

S3 Fig Nucleotide sequence conservation in the *Twist1* promoter and exon 1 regions among vertebrates. Homology searches of the region of promoter and exon 1 in *Twist1* among seven species of vertebrates were performed with T-coffee, a Multiple Sequence Alignment Server (http://tcoffee.vital-it.ch/apps/tcoffee/index.html). A dense CGI from the promoter to entire exon 1 was found with the mouse and human UCSC Genome Browser (http://genome.ucsc.edu/), as shown in Fig 2A and 2C. The alignments are gradually colored as green (low), yellow (mild), light pink (moderate), and dark pink (high) according to the consistency scores with T-coffee. Black horizontal lines indicate Sp1 binging motifs predicted with TFBIND (http://tfbind.hgc.jp) and JASPAR (http://jaspar.binf.ku.dk). Blue arrowheads at the promoter indicate nearly the same MSP region in human *Twist1*, as previously reported [14, 15]. Black and red arrowheads indicate the regions examined by BS and MSP in mouse *Twist1*, respectively. Blue arrows indicate the regions examined by ChIP assay. The genomic DNA sequences of the seven species used in this study are aligned as follows: human (Genbank accession number NC_018918), monkey (Macaca nemestrina, XM_011731108), mouse (NM_011658), rat (NC_005105.4), bird (Taeniopygia guttata, NW_002198270), frog (Xenopus tropicalis, NW_004668239), and fish (Danio rerio, NC_007130).
(TIF)

S4 Fig *Twist1* methylation status in MDGC cells. Bisulfite sequencing of the regions from -1843 to -1306 (BS-a), and -764 to -509 (BS-b) was performed in Twist1 expression-positive MDGC-9 and -negative MDGC-3 cells.
(TIF)

S5 Fig Expression levels of *Suv39h1* and *Suv39h2* in normal gastric mucosa and GC tissues. RT-PCR analysis of *Suv39h1* and *Suv39h2*. Five normal gastric mucosae tissues from *Atp4b-Cre* ^*−*^; *Cdh1* ^*loxP/loxP*^; *Trp53* ^*loxP/loxP*^ mice and MDGC-3 cells were used. *Gapdh* was used as an internal control.
(TIF)

S6 Fig RT-PCR of *Twist1* expression in GC cells with mithramycin A treatment. Two MDGC and two human GC cell lines with *Twist1* expression were treated with 100nM mithramycin A for 24 hr. *Twist1* expression was decreased in these four cell lines after treatment with mithramycin A compared to in mock ones (M). *GAPDH* was used as an internal control.
(TIF)

S1 Table Primer sequences for PCR used in this study. (XLSX)

S2 Table Primer sequences for MSP, BS-Seq, ChIP of *Twist1* used in this study. (XLSX)

## Abbreviations

BS: bisulfite sequencing
CGI: CpG island
ChIP: chromatin immunoprecipitation
DCKO: double conditional knockout mouse
DGC: diffuse-type gastric cancer
GAPDH: glyceraldehyde-3-phosphate dehydrogenase
GC: gastric cancer
H3K4me3: histone H3 lysine 4 tri-methylation
H3K9me3: histone H3 lysine 9 tri-methylation
H3K27me3: histone H3 lysine 27 tri-methylation
MSP: methylation-specific polymerase chain reaction
RT-PCR: Reverse transcription-polymerase chain reaction
qRT-PCR: quantitative RT-PCR
TSGs: tumor suppressor genes
TSS: transcript start site
5-aza-dC: 5-aza-2’-deoxycitidine

## References

[1] Barnes RM, Firulli AB. A twist of insight the role of Twist-family bHLH factors in development. Int J Dev Biol. 2009; 53: 909–924. 10.1387/ijdb.082747rb 19378251 PMC2737731

[2] Thisse B, Stoetzel C, Gorostiza-Thisse C, Perrin-Schmitt F. Sequence of the twist gene and nuclear localization of its protein in endomesodermal cells of early Drosophila embryos. EMBO J. 1988; 7: 2175–2183. 3416836 10.1002/j.1460-2075.1988.tb03056.x PMC454534

[3] Soo K, O’Rourke MP, Khoo PL, Steiner KA, Wong N, Behringer RR, et al Twist function is required for the morphogenesis of the cephalic neural tube and the differentiation of the cranial neural crest cells in the mouse embryo. Dev Biol. 2002; 247: 251–270. 12086465 10.1006/dbio.2002.0699

[4] Qin Q, Xu Y, He T, Qin C, Xu J. Normal and disease-related biological functions of Twist1 and underlying molecular mechanisms. Cell Res. 2012; 22: 90–106. 10.1038/cr.2011.144 21876555 PMC3351934

[5] Tsai JH, Donaher JL, Murphy DA, Chau S, Yang J. Spatiotemporal regulation of epithelial-mesenchymal transition is essential for squamous cell carcinoma metastasis. Cancer Cell. 2012; 22: 725–736. 10.1016/j.ccr.2012.09.022 23201165 PMC3522773

[6] Yang J, Mani SA, Donaher JL, Ramaswamy S, Itzykson RA, Come C, et al Twist, a master regulator of morphogenesis, plays an essential role in tumor metastasis. Cell. 2004; 117: 927–939. 15210113 10.1016/j.cell.2004.06.006

[7] Portela A, Esteller M. Epigenetic modifications and human disease. Nat Biotechnol. 2010; 28: 1057–1068. 10.1038/nbt.1685 20944598

[8] Baylin SB, Jones PA. A decade of exploring the cancer epigenome—biological and translational implications. Nat Rev Cancer. 2011; 11: 726–734. 10.1038/nrc3130 21941284 PMC3307543

[9] Rodríguez-Paredes M, Esteller M. Cancer epigenetics reaches mainstream oncology. Nat Med. 2011; 17: 330–339. 10.1038/nm.2305 21386836

[10] Esteller M. Epigenetic gene silencing in cancer: the DNA hypermethylome. Hum Mol Genet. 2007; 16: R50–R59. 17613547 10.1093/hmg/ddm018

[11] Kang GH, Lee S, Cho NY, Gandamihardja T, Long TI, Weisenberger DJ, et al DNA methylation profiles of gastric carcinoma characterized by quantitative DNA methylation analysis. Lab Invest. 2008; 88: 161–170. 18158559 10.1038/labinvest.3700707

[12] Okada T, Suehiro Y, Ueno K, Mitomori S, Kaneko S, Nishioka M, et al TWIST1 hypermethylation is observed frequently in colorectal tumors and its overexpression is associated with unfavorable outcomes in patients with colorectal cancer. Genes Chromosomes Cancer. 2010; 49: 452–462. 10.1002/gcc.20755 20140954

[13] Kwon MJ, Kwon JH, Nam ES, Shin HS, Lee DJ, Kim JH, et al TWIST1 promoter methylation is associated with prognosis in tonsillar squamous cell carcinoma. Hum Pathol. 2013; 44: 1722–1729. 10.1016/j.humpath.2013.03.004 23664538

[14] Gort EH, Suijkerbuijk KP, Roothaan SM, Raman V, Vooijs M, van der Wall E, et al Methylation of the TWIST1 promoter, TWIST1 mRNA levels, and immunohistochemical expression of TWIST1 in breast cancer. Cancer Epidemiol Biomarkers Prev. 2008; 17: 3325–3330. 10.1158/1055-9965.EPI-08-0472 19064546

[15] Ruppenthal RD, Nicolini C, Filho AF, Meurer R, Damin AP, Rohe A, et al TWIST1 promoter methylation in primary colorectal carcinoma. Pathol Oncol Res. 2011; 17: 867–872. 10.1007/s12253-011-9395-6 21461979

[16] Takahashi T, Saikawa Y, Kitagawa Y. Gastric cancer: current status of diagnosis and treatment. Cancers (Basel). 2013; 5: 48–63. 24216698 10.3390/cancers5010048 PMC3730304

[17] Yuasa Y. Control of gut differentiation and intestinal-type gastric carcinogenesis. Nat Rev Cancer. 2003; 3: 592–600. 12894247 10.1038/nrc1141

[18] Dicken BJ, Bigam DL, Cass C, Mackey JR, Joy AA, Hamilton SM. Gastric adenocarcinoma: review and considerations for future directions. Ann Surg. 2005; 241: 27–39. 15621988 10.1097/01.sla.0000149300.28588.23 PMC1356843

[19] Shimada S, Mimata A, Sekine M, Mogushi K, Akiyama Y, Fukamachi H, et al Synergistic tumour suppressor activity of E-cadherin and p53 in a conditional mouse model for metastatic diffuse-type gastric cancer. Gut. 2012; 61: 344–353. 10.1136/gutjnl-2011-300050 21865403

[20] Becker KF, Atkinson MJ, Reich U, Becker I, Nekarda H, Siewert JR, et al E-cadherin gene mutations provide clues to diffuse type gastric carcinomas. Cancer Res. 1994; 54: 3845–3852. 8033105

[21] Ranzani GN, Luinetti O, Padovan LS, Calistri D, Renault B, Burrel M, et al p53 gene mutations and protein nuclear accumulation are early events in intestinal type gastric cancer but late events in diffuse type. Cancer Epidemiol Biomarkers Prev. 1995; 4: 223–231. 7606196

[22] Rosivatz E, Becker I, Specht K, Fricke E, Luber B, Busch R, et al Differential expression of the epithelial-mesenchymal transition regulators snail, SIP1, and twist in gastric cancer. Am J Pathol. 2002; 161: 1881–1891. 12414534 10.1016/S0002-9440(10)64464-1 PMC1850763

[23] Zhang YQ, Guo XY, Han S, Chen Y, Ge FL, Bai FH, et al Expression and significance of TWIST basic helix-loop-helix protein over-expression in gastric cancer. Pathology. 2007; 39: 470–475. 17886095 10.1080/00313020701570053

[24] Yanagihara K, Tanaka H, Takigahira M, Ino Y, Yamaguchi Y, Toge T, et al Establishment of two cell lines from human gastric scirrhous carcinoma that possess the potential to metastasize spontaneously in nude mice. Cancer Sci. 2004; 95: 575–582. 15245593 10.1111/j.1349-7006.2004.tb02489.x PMC11159459

[25] van Engeland M, Weijenberg MP, Roemen GM, Brink M, de Bruïne AP, Goldbohm RA, et al Effects of Dietary Folate and Alcohol Intake on Promoter Methylation in Sporadic Colorectal Cancer: The Netherlands Cohort Study on Diet and Cancer. Cancer Res. 2003; 63: 3133–3177. 12810640

[26] Vuillemenot BR, Pulling LC, Palmisano WA, Hutt JA, Belinsky SA. Carcinogen exposure differentially modulates RAR-beta promoter hypermethylation, an early and frequent event in mouse lung carcinogenesis. Carcinogenesis. 2004; 25: 623–629. 14656941 10.1093/carcin/bgh038

[27] Chen R, Zhuge X, Huang Z, Lu D, Ye X, Chen C, et al Analysis of SEMA3B methylation and expression patterns in gastric cancer tissue and cell lines. Oncol Rep. 2014; 3: 1211–1218. 10.3892/or.2014.2972 24402303

[28] Varier RA, Timmers HT. Histone lysine methylation and demethylation pathways in cancer. Biochim Biophys Acta. 2011; 1815: 75–89. 10.1016/j.bbcan.2010.10.002 20951770

[29] Tian HP, Lun SM, Huang HJ, He R, Kong PZ, Wang QS, et al DNA methylation affects the SP1-regulated transcription of forkhead box F2 in breast cancer Cells. J Biol Chem. 2015; 290: 19173–19183. 10.1074/jbc.M114.636126 26070560 PMC4521039

[30] Mulero-Navarro S, Carvajal-Gonzalez JM, Herranz M, Ballestar E, Fraga MF, Ropero S, et al The dioxin receptor is silenced by promoter hypermethylation in human acute lymphoblastic leukemia through inhibition of Sp1 binding. Carcinogenesis. 2006; 27: 1099–1104. 16410262 10.1093/carcin/bgi344

[31] Choi ES, Nam JS, Jung JY, Cho NP, Cho SD. Modulation of specificity protein 1 by mithramycin A as a novel therapeutic strategy for cervical cancer. Sci Rep. 2014; 4: 7162 10.1038/srep07162 25418289 PMC4241519

[32] Sleiman SF, Langley BC, Basso M, Berlin J, Xia L, et al Mithramycin is a gene-selective Sp1 inhibitor that identifies a biological intersection between cancer and neurodegeneration. J Neurosci. 2011; 31: 6858–6870. 10.1523/JNEUROSCI.0710-11.2011 21543616 PMC3717375

[33] Rao X, Evans J, Chae H, Pilrose J, Kim S, Yan P, et al CpG island shore methylation regulates caveolin-1 expression in breast cancer. Oncogene. 2013; 32: 4519–4528. 10.1038/onc.2012.474 23128390 PMC3787796

[34] Otsubo T, Akiyama Y, Yanagihara K, Yuasa Y. SOX2 is frequently downregulated in gastric cancers and inhibits cell growth through cell-cycle arrest and apoptosis. Br J Cancer. 2008; 98: 824–831. 10.1038/sj.bjc.6604193 18268498 PMC2259184

[35] Douglas DB, Akiyama Y, Carraway H, Belinsky SA, Esteller M, Gabrielson E, et al Hypermethylation of a small CpGuanine-rich region correlates with loss of activator protein-2alpha expression during progression of breast cancer. Cancer Res. 2004; 64: 1611–1620. 14996719 10.1158/0008-5472.can-0318-2

[36] Brandeis M, Frank D, Keshet I, Siegfried Z, Mendelsohn M, Nemes A, et al Sp1 elements protect a CpG island from de novo methylation. Nature, 1994, 29; 6496: 435–458. 10.1038/371435a0 8090226

[37] Erfurth FE, Popovic R, Grembecka J, Cierpicki T, Theisler C, Xia ZB, et al MLL protects CpG clusters from methylation within the Hoxa9 gene, maintaining transcript expression. Proc Natl Acad Sci USA. 2008; 105: 7517–7522. 10.1073/pnas.0800090105 18483194 PMC2396713

[38] Kondo Y, Shen L, Issa JP. Critical role of histone methylation in tumor suppressor gene silencing in colorectal cancer. Mol Cell Biol. 2003; 23: 206–215. 12482974 10.1128/MCB.23.1.206-215.2003 PMC140684

[39] Ezponda T, Popovic R, Shah MY, Martinez-Garcia E, Zheng Y, Min DJ, et al The histone methyltransferase MMSET/WHSC1 activates TWIST1 to promote an epithelial-mesenchymal transition and invasive properties of prostate cancer. Oncogene. 2013; 32: 2882–2890. 10.1038/onc.2012.297 22797064 PMC3495247

[40] Cai L, Ma X, Huang Y, Zou Y, Chen X. Aberrant histone methylation and the effect of Suv39H1 siRNA on gastric carcinoma. Oncol Rep. 2014; 31: 2593–2600. 10.3892/or.2014.3135 24737085

[41] Sone K, Piao L, Nakakido M, Ueda K, Jenuwein T, Nakamura Y, et al Critical role of lysine 134 methylation on histone H2AX for γ-H2AX production and DNA repair. Nat Commun. 2014; 5: 5691 10.1038/ncomms6691 25487737 PMC4268694

[42] Lachner M, O'Carroll D, Rea S, Mechtler K, Jenuwein T. Methylation of histone H3 lysine 9 creates a binding site for HP1 proteins. Nature. 2001; 410: 116–120. 11242053 10.1038/35065132

[43] Dong C, Wu Y, Wang Y, Wang C, Kang T, Rychahou PG, et al Interaction with Suv39H1 is critical for Snail-mediated E-cadherin repression in breast cancer. Oncogene. 2013; 32: 1351–1362. 10.1038/onc.2012.169 22562246 PMC3703513

[44] Benlhabib H, Mendelson CR. Epigenetic regulation of surfactant protein A gene (SP-A) expression in fetal lung reveals a critical role for Suv39h methyltransferases during development and hypoxia. Mol Cell Biol. 2011; 31: 1949–1958. 10.1128/MCB.01063-10 21402781 PMC3133366

[45] O'Carroll D, Scherthan H, Peters AH, Opravil S, Haynes AR, Laible G, et al Isolation and characterization of Suv39h2, a second histone H3 methyltransferase gene that displays testis-specific expression. Mol Cell Biol. 2000; 20: 9423–9433. 11094092 10.1128/mcb.20.24.9423-9433.2000 PMC102198

[46] Suske G. The Sp-family of transcription factors. Gene. 1999; 238: 291–300. 10570957 10.1016/s0378-1119(99)00357-1

[47] Ohkuma M, Funato N, Higashihori N, Murakami M, Ohyama K, Nakamura M. Unique CCT repeats mediate transcription of the TWIST1 gene in mesenchymal cell lines. Biochem Biophys Res Commun. 2007; 352: 925–931. 17157810 10.1016/j.bbrc.2006.11.114

[48] Gong M, Yu W, Pei F, You J, Cui X, McNutt MA, et al. KLF6/Sp1 initiates transcription of the tmsg-1 gene in human prostate carcinoma cells: an exon involved mechanism. J Cell Biochem. 2012; 113: 329–339. 10.1002/jcb.23359 21928351

[49] Zheng JB, Zhou YH, Maity T, Liao WS, Saunders GF. Activation of the human PAX6 gene through the exon 1 enhancer by transcription factors SEF and Sp1. Nucleic Acids Res. 2001; 29: 4070–4078. 11574690 10.1093/nar/29.19.4070 PMC60230

[50] Zhu WG, Srinivasan K, Dai Z, Duan W, Druhan LJ, Ding H, et al Methylation of adjacent CpG sites affects Sp1/Sp3 binding and activity in the p21 (Cip1) promoter. Mol Cell Biol. 2003; 23: 4056–4065. 12773551 10.1128/MCB.23.12.4056-4065.2003 PMC156121

[51] Wang L, Wei D, Huang S, Peng Z, Le X, Wu TT, et al Transcription factor Sp1 expression is a significant predictor of survival in human gastric cancer. Clin Cancer Res. 2003; 9: 6371–6380. 14695137

[52] Hsu TI, Wang MC, Chen SY, Yeh YM, Su WC, Chang WC, et al Sp1 expression regulates lung tumor progression. Oncogene. 2012; 31: 3973–3988. 10.1038/onc.2011.568 22158040 PMC3432230

[53] Japanese Gastric Cancer Association. Japanese classification of gastric carcinoma: 3rd English edition. Gastric Cancer. 2011; 14:101–112. 10.1007/s10120-011-0041-5 21573743

