# Supplementary figures and images for: DNA Methylation in the Exon 1 Region and Complex Regulation of Twist1 Expression in Gastric Cancer Cells
Source: PLoS One. 2015 Dec 22;10(12):e0145630. doi: 10.1371/journal.pone.0145630 (PMC4687923; doi:10.1371/journal.pone.0145630)

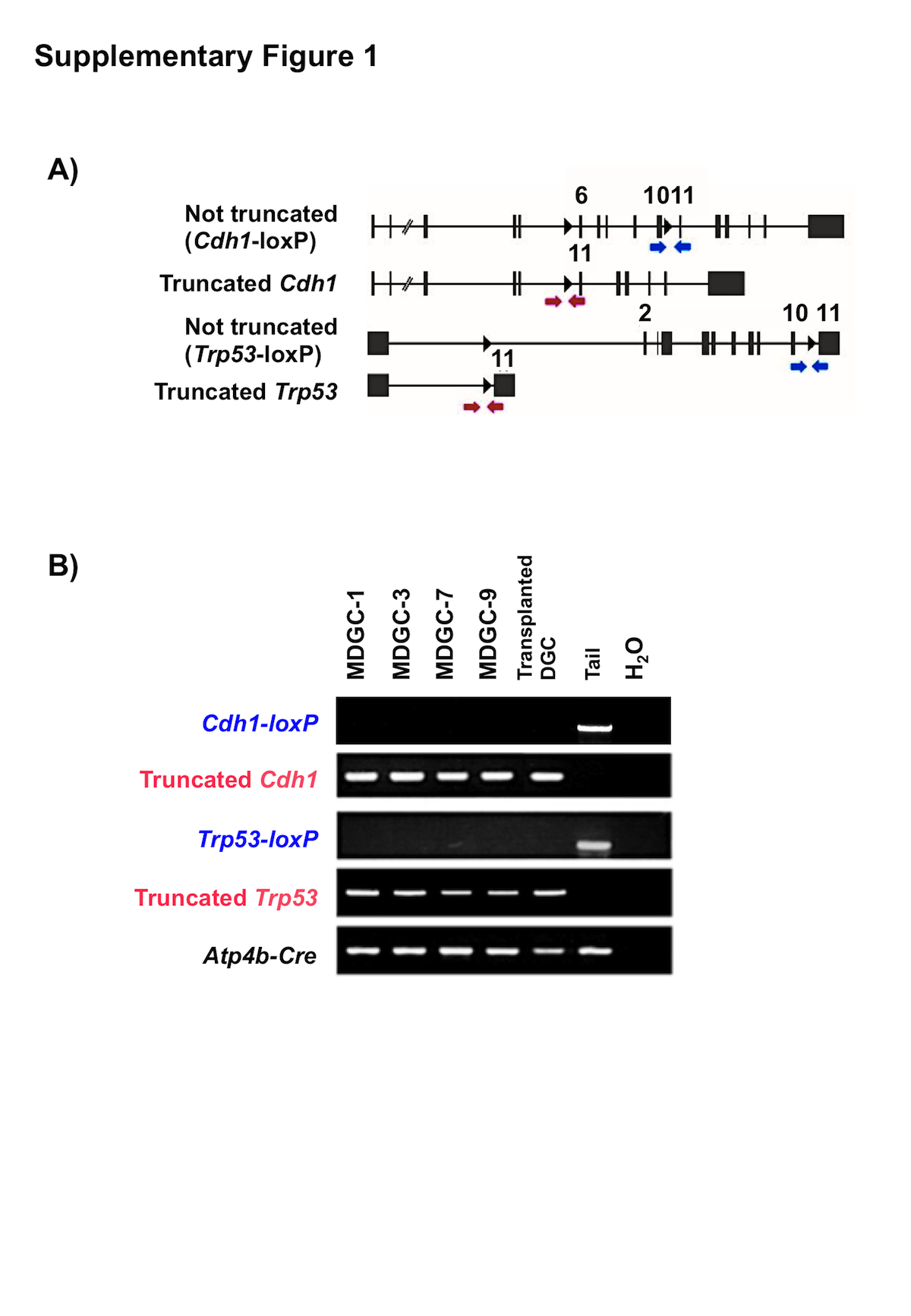

Supplement: S1 Fig — (A) Schematic representation of the genomic structures of Trp53 sequences and their truncated regions. The filled boxes denote the exon. The blue and pink arrows indicate the regions examined by RT-PCR. The numbers indicate exon numbers. (B) Genomic PCR analysis of truncated Cdh1 and Trp53 alleles in MDGC cells. DNA was amplified by PCR using primer, shown in S1 Table. DGC tumors from DCKO mice were transplanted to nude mice. Genomic DNA extracted from the transplanted DGC and DCKO mouse tails was used as positive controls for the truncated and not-truncated alleles of these two genes, respectively. Atp4b-Cre was used as an internal control. (TIF) [file pone.0145630.s001.tif]

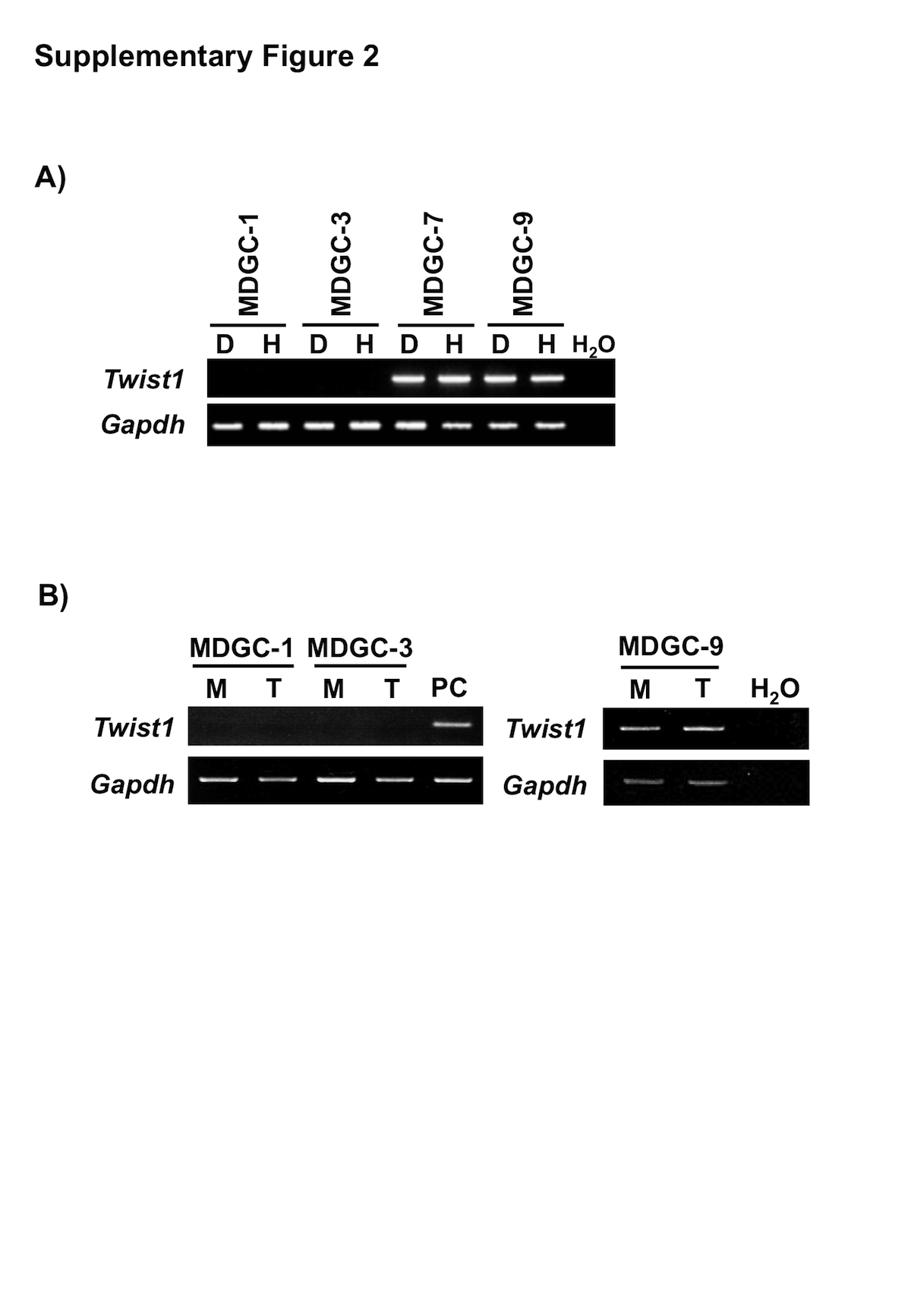

Supplement: S2 Fig — (A) RT-PCR analysis of The Twist1 mRNA expression in GC cells. Twist1 expression was not changed when the cells cultured in different mediums. D, Dulbecco’s modified Eagle’s medium containing high glucose supplemented with 10% fetal bovine serum; H, Ham’s F12 supplemented with 5% horse serum. (B) The Twist1 mRNA levels did not change after treatment with TSA in Twist1 expression-positive (MDGC-9) and -negative (MDGC-1, MDGC-3) cells. M, mock; T, TSA. (TIF) [file pone.0145630.s002.tif]

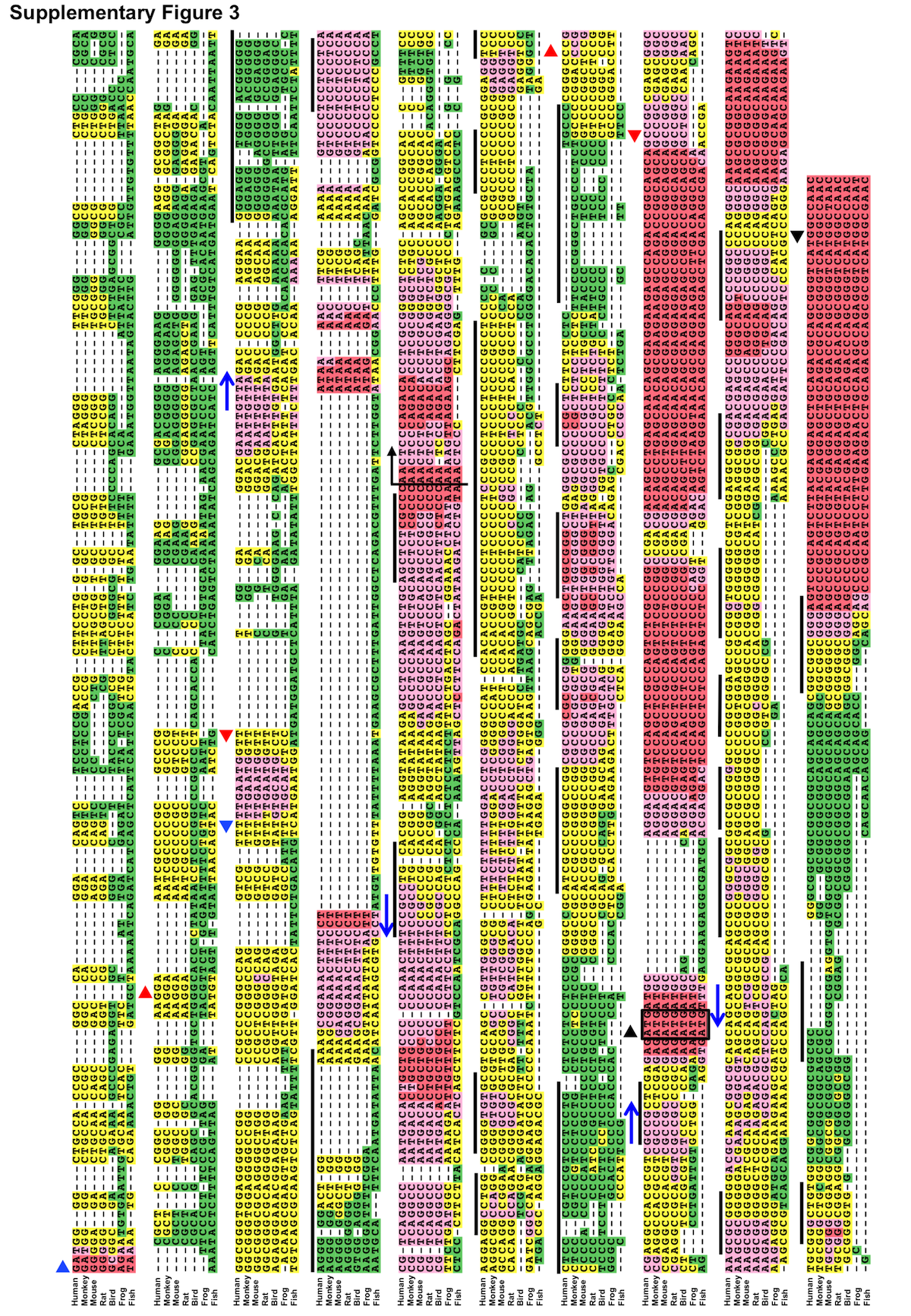

Supplement: S3 Fig — Homology searches of the region of promoter and exon 1 in Twist1 among seven species of vertebrates were performed with T-coffee, a Multiple Sequence Alignment Server (http://tcoffee.vital-it.ch/apps/tcoffee/index.html). A dense CGI from the promoter to entire exon 1 was found with the mouse and human UCSC Genome Browser (http://genome.ucsc.edu/), as shown in Fig 2A and 2C. The alignments are gradually colored as green (low), yellow (mild), light pink (moderate), and dark pink (high) according to the consistency scores with T-coffee. Black horizontal lines indicate Sp1 binging motifs predicted with TFBIND (http://tfbind.hgc.jp) and JASPAR (http://jaspar.binf.ku.dk). Blue arrowheads at the promoter indicate nearly the same MSP region in human Twist1, as previously reported [14, 15]. Black and red arrowheads indicate the regions examined by BS and MSP in mouse Twist1, respectively. Blue arrows indicate the regions examined by ChIP assay. The genomic DNA sequences of the seven species used in this study are aligned as follows: human (Genbank accession number NC_018918), monkey (Macaca nemestrina, XM_011731108), mouse (NM_011658), rat (NC_005105.4), bird (Taeniopygia guttata, NW_002198270), frog (Xenopus tropicalis, NW_004668239), and fish (Danio rerio, NC_007130). (TIF) [file pone.0145630.s003.tif]

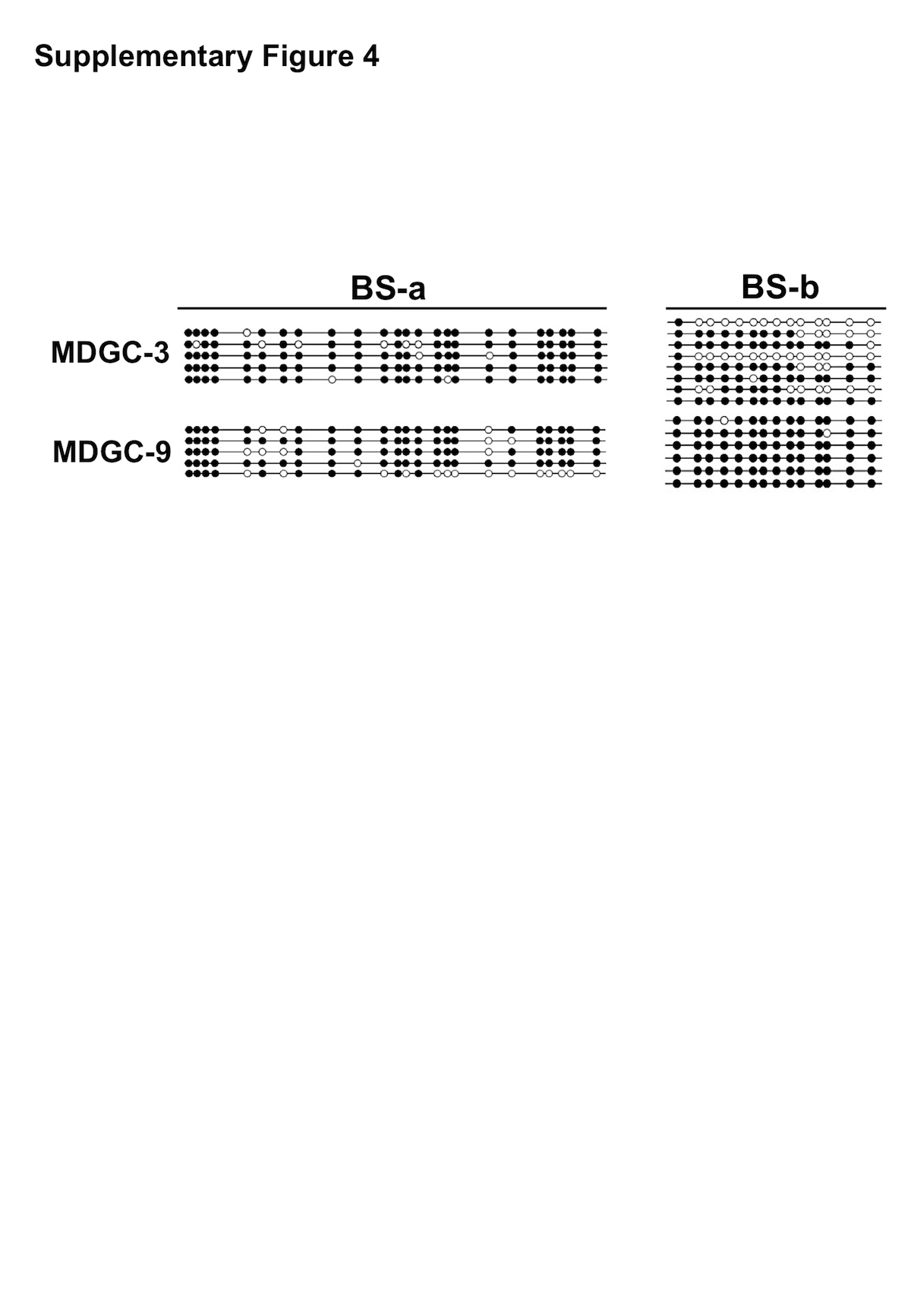

Supplement: S4 Fig — Bisulfite sequencing of the regions from -1843 to -1306 (BS-a), and -764 to -509 (BS-b) was performed in Twist1 expression-positive MDGC-9 and -negative MDGC-3 cells. (TIF) [file pone.0145630.s004.tif]

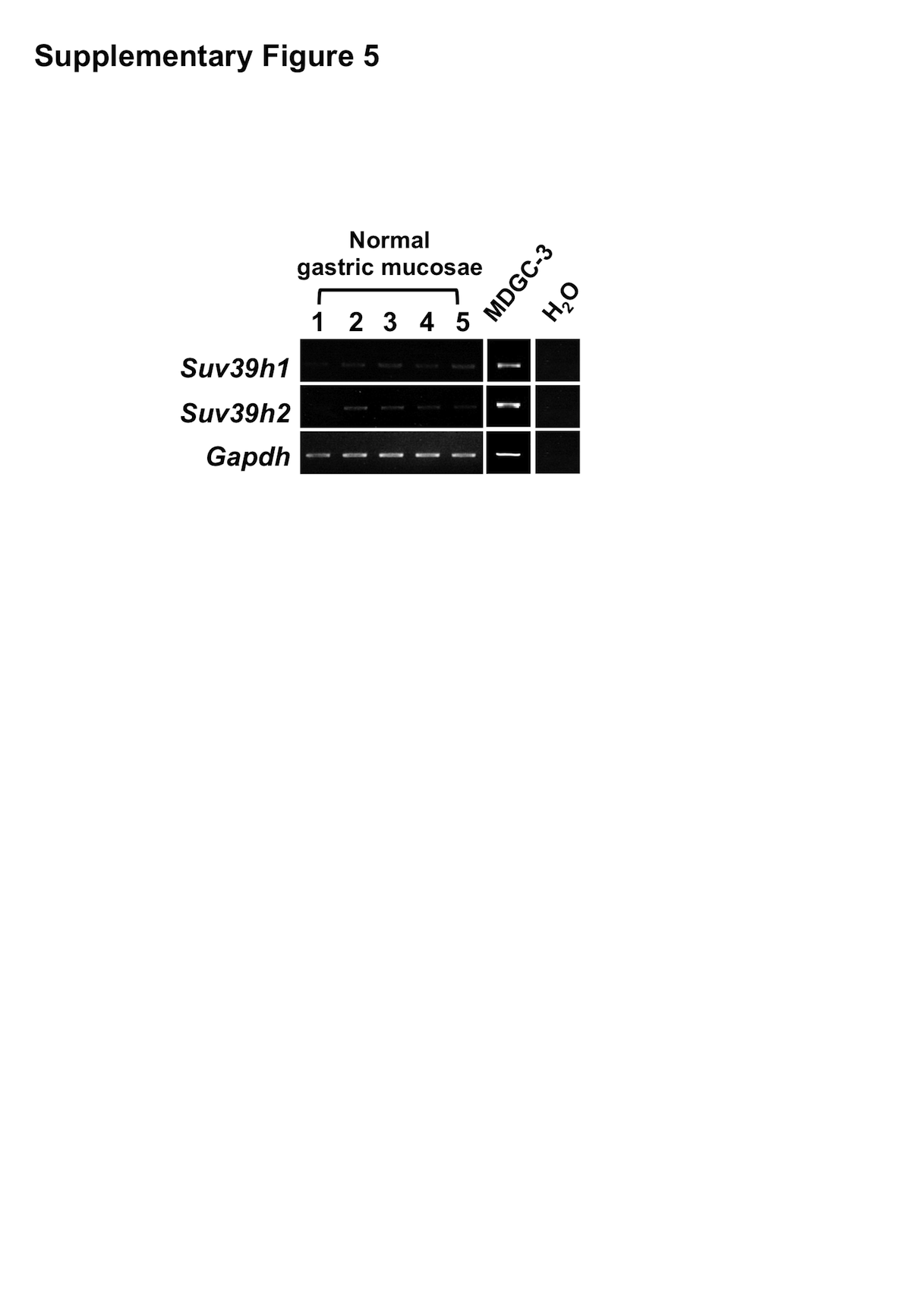

Supplement: S5 Fig — RT-PCR analysis of Suv39h1 and Suv39h2. Five normal gastric mucosae tissues from Atp4b-Cre −; Cdh1 loxP/loxP; Trp53 loxP/loxP mice and MDGC-3 cells were used. Gapdh was used as an internal control. (TIF) [file pone.0145630.s005.tif]

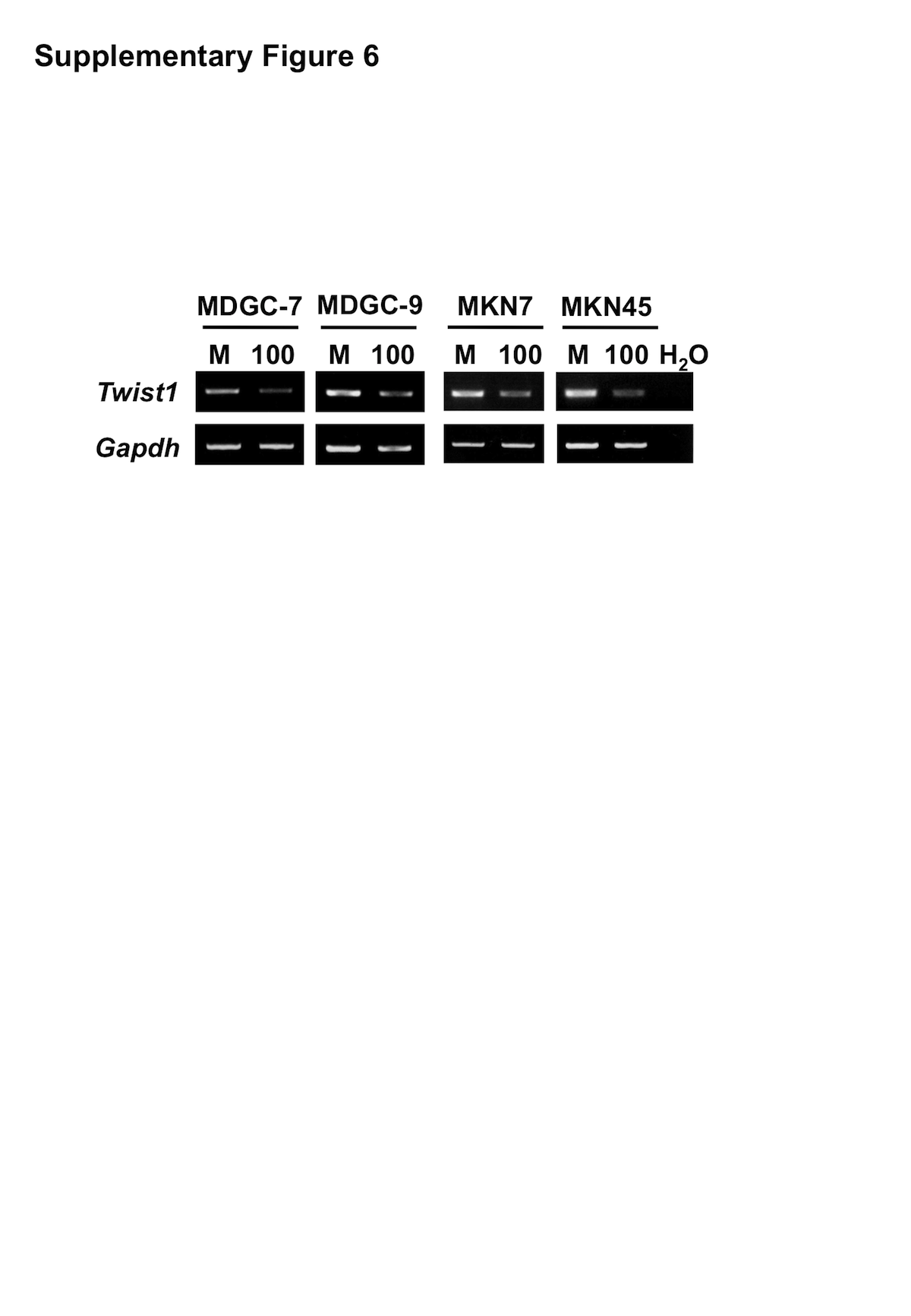

Supplement: S6 Fig — Two MDGC and two human GC cell lines with Twist1 expression were treated with 100nM mithramycin A for 24 hr. Twist1 expression was decreased in these four cell lines after treatment with mithramycin A compared to in mock ones (M). GAPDH was used as an internal control. (TIF) [file pone.0145630.s006.tif]
